# Design and impact evaluation of a digital reproductive health program in Rwanda using a cluster randomized design: study protocol

**DOI:** 10.1186/s12889-020-09746-7

**Published:** 2020-11-13

**Authors:** Cara Nolan, Laura Packel, Rebecca Hope, Jordan Levine, Laura Baringer, Emmyson Gatare, Aline Umubyeyi, Felix Sayinzoga, Michael Mugisha, Janepher Turatsinze, Aimee Naganza, Laiah Idelson, Stefano Bertozzi, Sandra McCoy

**Affiliations:** 1 School of Public Health, University of California, Berkeley, 2121 Berkeley Way West, MC 7360, Berkeley, CA 94720 USA; 2 YLabs, 2nd Floor, Golden Plaza, KG 546 St. Kacyiru, Kigali, Rwanda; 3 Department of Epidemiology and Biostatistics, School of Public Health, College of Medicine and Health Sciences, University of Rwanda, P.O. Box: 5229, Kigali, Rwanda; 4 Rwanda Biomedical Center, KG 17 Ave, towards Amahoro Stadium, Remera, Rukiri II, Remera, Gasabo, Kigali, Rwanda; 5 Society for Family Health, Rwanda, Plot 99 KG543 St. Kacyiru, PO Box: 3040, Kigali, Rwanda; 6 YTH Initiative, ETR, 1630 San Pablo Avenue, Suite 500, Oakland, CA 94612 USA

**Keywords:** Human-centered design, Cluster randomized controlled trial, Adolescent sexual and reproductive health, Family planning and reproductive health, Rwanda, Digital health, Hybrid type 2 effectiveness-implementation study, Uptake of modern contraceptive methods

## Abstract

**Background:**

Rwandan adolescents have limited access to high-quality family planning and reproductive health (FP/RH) information and care to prevent unplanned pregnancy and HIV/STIs. In addition to the immediate implications for health and well-being, teenage pregnancy is a significant cause of school drop-out, limiting girls’ future potential and employment opportunities. This study introduces a direct-to-consumer digital education program that uses storytelling to deliver age-appropriate FP/RH information and economic empowerment training to adolescents. It also facilitates access to high-quality, youth-friendly FP/RH care and products. We evaluate two different school-based models of its implementation to understand how to optimize the uptake of contraception and HIV testing among adolescents.

**Methods:**

The study consists of two distinct phases. The first formative intervention design phase, conducted from 2016 to 2019, used a human-centered design methodology to develop the intervention alongside over 600 Rwandan adolescents, their parents, teachers, and healthcare providers. Through this methodology, we sought to maximize the fit between evidence-based practices (uptake of modern contraception and HIV testing) and the implementation context of adolescents in Rwanda. The second phase is an impact evaluation, in which we will use a Hybrid Trial Type 2 Effectiveness-Implementation study design to determine the overall effectiveness of this digital intervention as well as the relative effectiveness of the two different school-based implementation models. This takes the form of a 3-arm cluster-randomized non-inferiority trial, with a sample of 6000 youth aged 12–19 in 60 schools across 8 districts in Rwanda. Primary outcome measures include use of modern contraception, delayed initiation of childbearing, and uptake of HIV testing.

**Discussion:**

This study will yield insights into not only whether this digital intervention is successful in achieving the intended sexual and reproductive health outcomes, but also which mechanisms are likely to drive this effectiveness. The methodologies used are broadly applicable to the design, implementation, and evaluation of other behavior-based health programs in low and middle-income countries.

**Trial registration:**

ClinicalTrials.gov Identifier: NCT04198272. Prospectively registered 13 December 2019.

**Supplementary Information:**

The online version contains supplementary material available at 10.1186/s12889-020-09746-7.

## Background

Adolescents in Rwanda have limited access to high-quality family planning and reproductive health (FP/RH) information and care to prevent unplanned pregnancy and HIV/STIs. In the most recent Rwanda Demographic and Health Survey (DHS), only 12% of sexually active unmarried women ages 15–19 report currently using contraception [1], likely due to a multitude of barriers including economic constraints, incorrect perception of risk, misinformation, and harmful social and cultural norms around adolescent sexuality. Consequently, one in five girls aged 19 have begun bearing children [1]. In addition to the immediate implications on health and well-being, teenage pregnancy is a significant cause of school drop-out, which limits girls’ future potential and employment opportunities [2].

Though the government recognizes the right of young people to access FP/RH information and care [3], previous efforts to deliver school-based sexual health education in Rwanda have been of limited effectiveness [4]. Concurrently, there is evidence that urban youth in East Africa increasingly use the internet and social media to access information about sex and contraception, which has the potential to meet these information needs [5]. However, the quality of this information can be inconsistent [6]. Thus, the environment is prime to pursue a digital intervention to provide FP/RH information to adolescents in Rwanda. This protocol describes the approaches that have been used to develop one such intervention, CyberRwanda, as well as the plan for rigorously evaluating its impact.

CyberRwanda is a digital intervention for urban and peri-urban youth aged 12–19. Designed in partnership with youth and their communities, it combines age-appropriate storytelling content about FP/RH and economic empowerment, with discreet, streamlined access to contraception and other essential products from trained pharmacists through a mobile ordering platform. Through CyberRwanda, young people can order products online for discreet in-person collection at participating pharmacies, after answering medically-relevant questions and reading information about the products online. All participating pharmacies are trained on the provision of youth-friendly care. CyberRwanda addresses the challenges adolescents face in finding and accessing accurate information on socially taboo topics, locating youth-friendly care, and then discreetly ordering and receiving products. Co-designed with healthcare providers, accompanying training tools for pharmacists support them to provide youth-friendly care.

The objective of CyberRwanda is to improve a spectrum of adolescent health outcomes, with specific focus on increased uptake of modern contraception, delayed initiation of childbearing, and increased rates of HIV testing. Within a Theory of Planned Behavior (TPB) framework [7], we hypothesize that CyberRwanda will strengthen the knowledge, attitudes, perceived social norms, and perceived behavioral control of participants, leading to the aforementioned health outcomes.

Starting in 2020, CyberRwanda will be implemented in schools using two different models: self service and facilitated. In both the models, participating schools will be provided with tablets and hotspots to access CyberRwanda. Within each school, the CyberRwanda implementation team will work with select teachers, administrators and students to enable access to the tablets. In the facilitated model, selected students are also trained as “peer facilitators” to deliver weekly activities to complement the online content. The aim of using two implementation models is to determine whether digital health education programs require face-to-face peer or facilitator support in order to be effective, or whether the knowledge and behavior change components can be achieved through the online components only. Although there exist a number of systematic reviews of digital media interventions for sexual health promotion [6], there is limited evidence on the factors related to successful implementation and the relative effectiveness of web-based vs. face-to-face delivery mechanisms [6, 8, 9]. Our evaluation will test the comparative effectiveness of online-only and online-*plus*-face-to-face delivery models.

We will evaluate the impact of CyberRwanda through a 3-arm cluster-randomized non-inferiority trial. By comparing two different methods of school-based implementation of CyberRwanda and a control group which receives standard available services in the community, we are using a Type 2 Effectiveness-Implementation Hybrid study design [10], in which we simultaneously assess overall effectiveness as well as effectiveness of either implementation model relative to one another. We draw upon Proctor’s Implementation Science framework to understand why, and in what context, CyberRwanda is or is not effective.

## Methods

The study consists of two distinct phases. The first phase, conducted from 2016 to 2019, is the intervention design phase in which we used a human-centered design methodology consisting of small group discussions, structured interviews, and rapid prototyping of intervention elements with over 600 adolescents and other stakeholders to determine the most effective and appropriate design for the intervention. The second phase, to be conducted from 2020 onwards, consists of the evaluation of the intervention in a controlled research design. First, we present the methodology of the intervention design phase and the insights gained from the different elements therein, leading to the final design of the intervention. Second, we present the protocol for the impact evaluation.

### Phase 1: intervention design

Over the period from July 2016–October 2018, we designed the CyberRwanda intervention using a human-centered design methodology. In a seminal piece in the Stanford Social Innovation Review (2015), Panthea Lee defines human-centered design as “a multi-stage problem-solving process that optimizes solutions based on users’ needs, behaviors, constraints, and operating contexts. Solutions are repeatedly tested and refined throughout the design and development process before implementation” [11]. The methodology allowed us to center the voice of young Rwandans and engage them to co-create insights and co-design the intervention, while also considering the broader system of stakeholders. We outline each of the major steps below.

### Phase 1.1 - design research (July 2016–July 2017)

Over the course of 12 months, we conducted three rounds of initial design research through interviews and small group discussions with 212 youth, parents, teachers and healthcare providers in urban Kigali, peri-urban Ruhango and peri-urban Butare to understand the experiences, needs, aspirations and barriers for adolescents regarding reproductive health and employment. We started with the parameters of seeking to design a digital education intervention to address these issues, and so initial design research sought to test the feasibility of a digital solution. A key consideration was designing to ensure equity of access for youth with and without access to technology, so a secondary focus of the design research was to map access to and use of technology, mobile devices, and social media among the target population.

Interviews and small group discussions (which typically comprised groups of 2–5 participants) were semi-structured in nature and followed a guide with a set of topics to cover. These sessions were led by the research team in Kinyarwanda or English (with Kinyarwanda translation if needed^1^) and typically ranged from 45 to 60 min. Unlike traditional qualitative research, which typically asks the same questions each session in order to build consistent insights between interviews [12], the questions asked in each interview, group activity, and discussion were iteratively refined between sessions to test hypotheses and validate insights derived from previous interviews. The objective was to understand the specific context, behavioral barriers, and behavioral drivers of our target user population in order to design a successful intervention. It was also used to validate emerging insights with participants. Building this understanding is a central tenet of human-centered design. Throughout each participant session in the design research, prototyping and user testing phases, written consent was provided by all participants over 18, and assent for all participants under the age of 18. Additional consent for youth under 18 to participate was provided by parents and caregivers, or teachers when sessions took place in schools.

The interactive format of each session included activities such as roleplay, crafting, and card sorting. Roleplay situations included, for example, participants acting out hypothetical interactions with health providers to reveal challenges they face in receiving care. Craft activities, such as collages, allowed participants to articulate abstract concepts such as their ‘ideal future’. Card sorting is a process in which participants rank cards with different images in order of preference. For example, in one card sorting exercise, cards showed the names of people in young people’s lives (teacher, mother, friend, priest etc.), and participants were asked to rank these in order of those they trust the most to those they trust the least. This built understanding of what inspires trust for young Rwandans and helped us incorporate these features into the characters and narratives on the digital platform. These techniques helped elucidate participants’ beliefs, behavioral preferences and experiences as they relate to FP/RH specifically, but also their broader experiences of everyday life, motivations, aspirations and challenges. This broader inquiry helped to uncover insights to improve the desirability and feasibility of potential design solutions.

Information gathered during initial design research was synthesized into insights that drove intervention development in a process standard for a human-centered design project. After each session, observations that related to the areas of research interest were recorded on sticky notes. Common observations across sessions were grouped together into key insights in a process of ‘affinity mapping’. Each subsequent interview or small group discussion was used to validate and refine the emerging insights. The set of insights (Table 1) were used to inform major design decisions, including a set of design principles that guided the development of potential solutions.

**Table 1:** Key Insights from Design Research Informing Major Design Decisions for a Potential Digital Platform

Insight	Design Decision
Youth prioritize employment and economic opportunities over health. Consistent with research findings from similar settings [13], ‘preparing for your future’ is a more culturally acceptable frame for the content than ‘learning about FP/RH’.	Include content on employment skills as well as health; introduce FP/RH content through character stories rather than as isolated FP/RH topics.
For unmarried youth, the stigma associated with accessing contraception makes the risk of exposure unbearable. Youth prefer pharmacists over clinics to access FP/RH advice and products due to their anonymity and more rapid service.	Shift focus from clinics to pharmacists; reduce barriers for youth to access appropriate care through pharmacies.
Pharmacists often lack the skills and knowledge to deliver youth-friendly care; some are afraid to provide contraception to adolescents due to cultural norms, personal biases or misunderstandings, and uncertainty around national policies on adolescent FP/RH.	Develop an intervention for pharmacists to increase their knowledge of FP/RH topics and national guidelines, and to deconstruct bias on provision of contraception to adolescents.
Urban youth report using smartphones to seek out information about FP/RH, and rates of smartphone usage among the target population are high, but computers are not readily available to the target population.	Design a web application that can be accessed via smartphone.
Adolescents consider home delivery of contraceptive products too risky and would prefer to pick up products from the pharmacy themselves.	Discard design options using motorcycle delivery in favor of a streamlined pharmacy collection option incorporating online ordering.
When choosing contraceptive methods, adolescents don’t know which products they need and may be afraid to ask too many questions.	Integrate education and context into the process of ordering contraceptive products online so youth are more confident in their choices.

Table 1 outlines the key insights that emerged from design research and subsequent rough prototyping, and how they influenced major design decisions

This process yielded a number of critical insights that informed the intervention design. For example, we learned that it was not just adolescent girls who wanted a trusted source of FP/RH information: boys also had limited knowledge about FP/RH and were often a primary driver of girls’ sexual decision-making. We therefore decided to expand our original scope to include boys as well as girls. Furthermore, fears of stigma and shame from community members presented a considerable barrier to accessing FP/RH care. Our initial assumption had been that a logical point of intervention would be in primary health clinics. In speaking with adolescents, we learned that they prefer to access contraceptive products through pharmacies rather than clinics, given that pharmacies offer faster and more confidential service. However, pharmacists were often reluctant to provide contraceptive products to unmarried adolescents. These findings informed a pivot towards a direct-to-consumer platform providing streamlined access to health products, supplied by a network of pharmacists, with signposting to clinics for longer-acting family planning and other reproductive health care.

Given the cultural and social sensitivity of topics concerning contraception and adolescent reproductive health, a key area of investigation in initial design research was how these messages would be received, and how to frame the intervention in a socially acceptable and constructive manner. During this phase, we talked to a wide range of parents, teachers and community leaders, including some religious leaders, to get their input on the concept and some early prototypes. We learned that certain ‘entry points’ into the conversation made the content more socially acceptable and well-received. For example, there is strong government support and prioritization for addressing teenage pregnancy in Rwanda as a cause of school dropout and ill health. Grounding the conversation in this government priority proved to be a productive framing.

### Phase 1.2 - prototyping: youth (September–October 2017) and provider (February–June 2018)

**Fig. 1:**
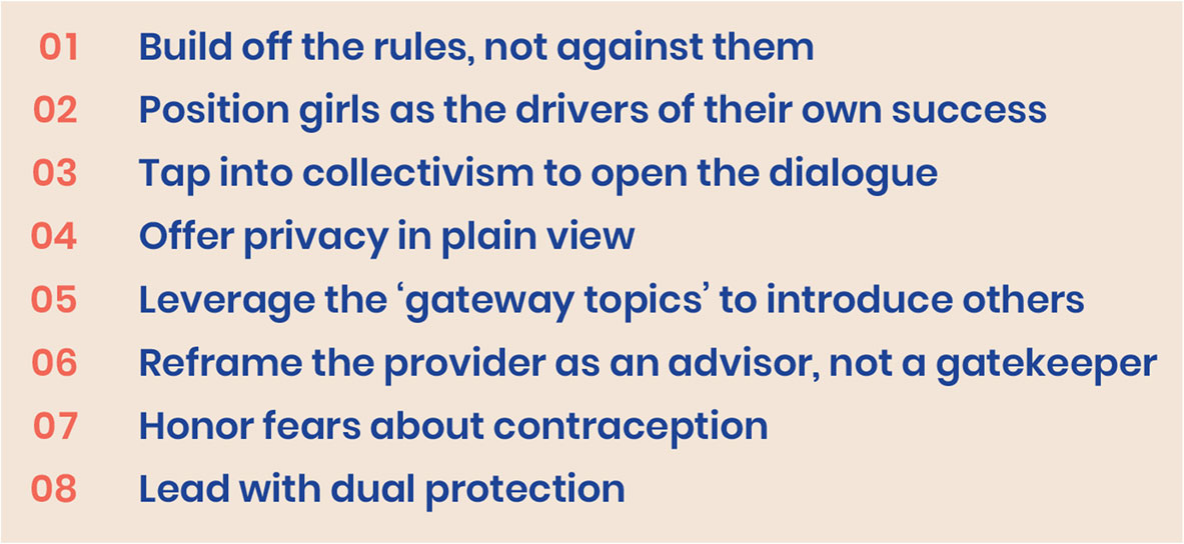
Design Principles. Design principles derived from initial design research and used to inform prototype development

**Fig. 2:**
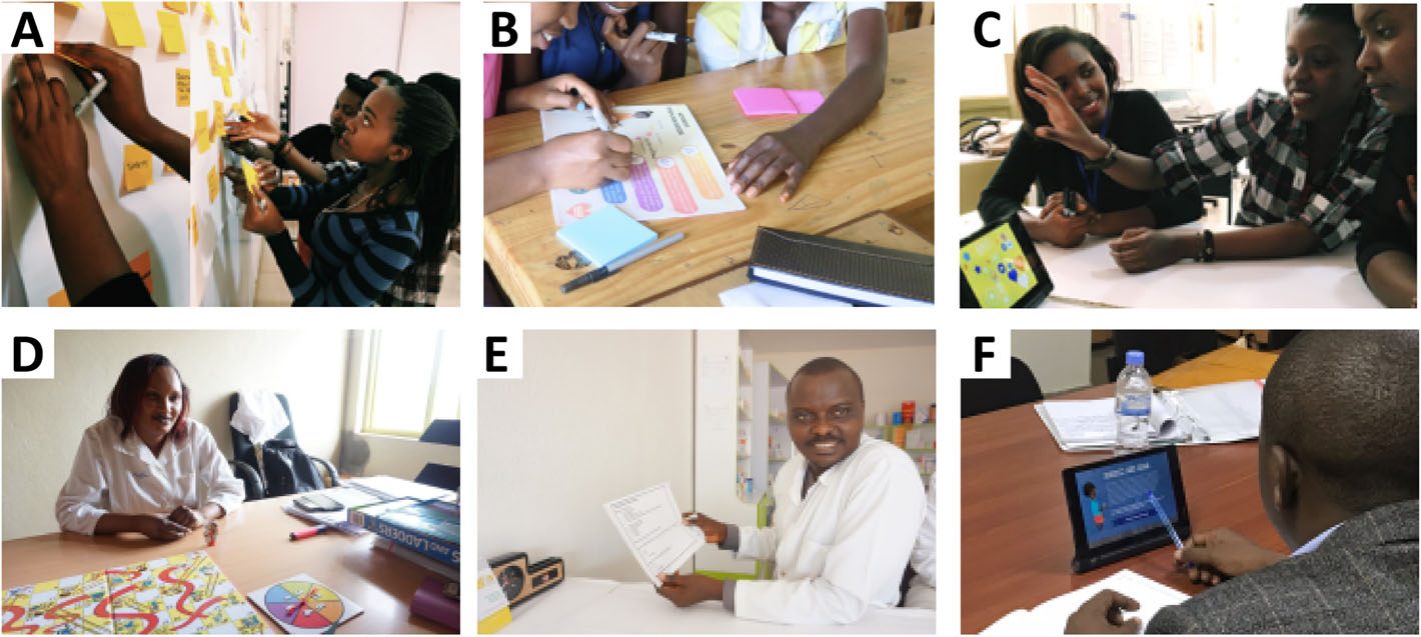
The Prototype Development Process. Steps involved in prototyping both youth (**a - c**) and provider-facing (**d - f**) solutions ranged from co-designing key elements of the solution to low- and high-fidelity testing of prototype solutions. **a**: Youth Co-Design - Mapping site content based on young people’s key questions; **b**: Youth Low-Fidelity Prototyping - Testing paper prototypes of content and visual design; **c**: Youth High-Fidelity Prototyping - Testing wireframe prototypes of the app; **d**: Provider Co-Design - Exploring gamified approaches to learning with providers; **e**: Provider Low-Fidelity Prototyping - Testing pre-screening methods to expedite care for youth; **f**: Provider High-Fidelity Prototyping - Testing an interactive digital prototype of the training game

The next phase of our process was prototyping. Prototyping involves bringing rough demonstrations of potential solutions to users to seek their feedback. The insights from initial design research enabled us to construct a set of design principles for the digital application (Fig. 1) from which to develop a set of rough prototype solutions (see select images of several prototypes in Fig. 2, and a full list of prototypes in Additional file 1). Design principles, such as “offer privacy in plain view” and “reframe the provider as an advisor, not gatekeeper”, offered guidance in terms of the type of solution, method of delivery, and messaging to use. Leveraging these principles, rough prototypes were developed that ranged from simple concepts to test (e.g. a discussion about whether motorcycle delivery of contraceptive products was appealing to adolescents), types of communication and key messages (e.g. paper-based hormonal diagrams and a “boy’s rulebook” that explains a man’s role in consent) through to more developed wireframes of the CyberRwanda website for users to interact with the characters, storylines and user interface. By having users react to a tangible idea in the form of a prototype, rather than abstract concepts, we were able to learn which solutions would be most successful, why, and in what format.

From September to October 2017, we tested a suite of rough prototypes through interactive design workshops with adolescents in urban Kigali and peri-urban Ruhango. Our sample included 31 girls aged 10–23 and 5 boys aged 15–17. Sessions included individual interviews and small group discussions. The group discussions were split according to age group (i.e. young adolescent vs. older adolescent), and facilitated in Kinyarwanda or in English with a Kinyarwanda translator when needed.

Nine prototypes were tested, including concepts such as “Ask Juliette”, a virtual nurse who answers questions and offers referrals to a local youth corner in a clinic; “Boy’s Rulebook”, a guide for boys to develop attitudes and knowledge about consent; and “Motorcycle delivery”, a proposal to deliver contraceptive products via motorbike. These prototypes tested the cultural acceptability, desirability and feasibility of education and service delivery channels. Each session began with the participants being presented with one of the prototypes to interact with. In the interactive prototyping workshops, participants were engaged through activities such as co-creation, in which participants made changes to the prototypes to reflect their needs and desires; journey maps, in which participants defined the key points in their experience; ranking, in which participants voted on their preferred prototypes or top concerns/priorities; and brainstorming, in which participants generated new ideas for prototypes to test. Following the interactive design workshops, the team refined the possible solutions into a narrower set of options to continue to develop. Observations from each prototyping session were recorded on sticky notes, and clustered into key themes as they related to different aspects of the prototype or intended behavioral change. After each subsequent session, similar observations were grouped together. If the same observation occurred more than once, the team considered it an important observation to respond to, and modified the prototype accordingly.

From February to June 2018, a second intensive round of prototyping was conducted. This phase had a focus on providers in addition to refining the youth-facing solutions selected from the first round of prototyping. Through 42 semi-structured interviews, 23 small group discussions and a co-design workshop, we engaged 63 providers, 4 youth coordinators, 12 boys and 33 girls in urban Kigali and peri-urban Huye. In two waves of prototyping, we developed 12 prototypes of increasingly high fidelity to test and refine ideas to reduce provider bias and improve quality in delivering reproductive health products and advice to adolescents.

The first wave of prototyping tested low fidelity mock-ups of the concepts such as paper prototypes showing the potential product interface and various interaction elements. Youth, parents, and healthcare providers gave feedback via interviews and small group discussions on these first-round prototypes. In response to their feedback and additional ideas, promising prototypes were further refined. In the second wave, high fidelity prototypes such as a digital mock-up of the pharmacist training game were presented to a different set of participants for their input. Participants were presented with the prototype and researchers gathered data through observing their interaction with it, including difficulties navigating the product and asking open-ended questions to elicit their reactions and gauge their understanding and engagement with it. Participants were also engaged in co-design during these sessions, suggesting new ideas and design elements to better engage users, such as the design of the product characters and the key categories of information needed to meet young people’s needs. Prototypes included, for example, a WhatsApp chat group among providers to discuss technical questions and learn from medical experts; a contraceptive cheat sheet that offered pharmacists rapid visual information for counselling youth on contraceptives; and a free daily SMS reminder for adolescents to take their oral contraceptive pill. From this phase of prototyping, we were able to refine the final solution’s function, content and aesthetic to develop into a software application.

### Phase 1.3 - product development and user testing (August 2018–August 2019)

Following design research and prototyping, we proceeded to develop our final intervention design in collaboration with a software development firm. Throughout this period, we engaged 237 users (85 boys, 87 girls, 23 parents or school administrators, and 51 pharmacists) in testing the emerging products through a combination of individual interviews and small group discussions, and developed a plan for implementing the products in schools and pharmacies. We iterated from low-fidelity prototypes such as paper mockups to realistic digital prototypes in order to incorporate user feedback on tone and voice of the content, visual design, characters and user flow through the website. We also considered how to improve user experience and reduce barriers to use. For example, we found that while many adolescents do not have their own smartphones, most have access to a device, and therefore we provided the option for youth to subscribe to SMS for a better order experience. In line with common practice in human-centered design, we ran multiple rounds of iterations in order to gauge users’ views on the look and feel of the products, and to ensure that they find the learning process fun, the content relevant, and the order process trustworthy.

### Phase 1.4 - content development and the theory of planned behavior (September–December 2019)

**Fig. 3:**
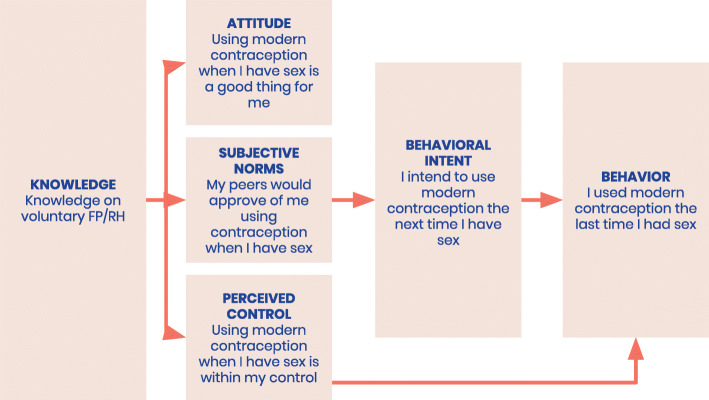
Behavioral pathway leading to increased contraceptive use. Example behavioral pathway defined by the Theory of Planned Behavior, from knowledge to behavior change in usage of modern contraceptive methods

In the final stages of developing the intervention, based on an Intervention Mapping protocol [14], we mapped each CyberRwanda behavioral outcome to the Theory of Planned Behavior (TPB). We then used those outcomes to develop the key messages, determine how best to communicate those messages throughout program content and design and guide the evaluation. As a commonly used tool in understanding health behaviors, the TPB explains how determinants of behavior include (1) intentions to engage in that behavior and (2) perceived control over that behavior [7]. Figure 3 outlines the behavioral pathways suggested by the framework.

### Final intervention design

**Fig. 4:**
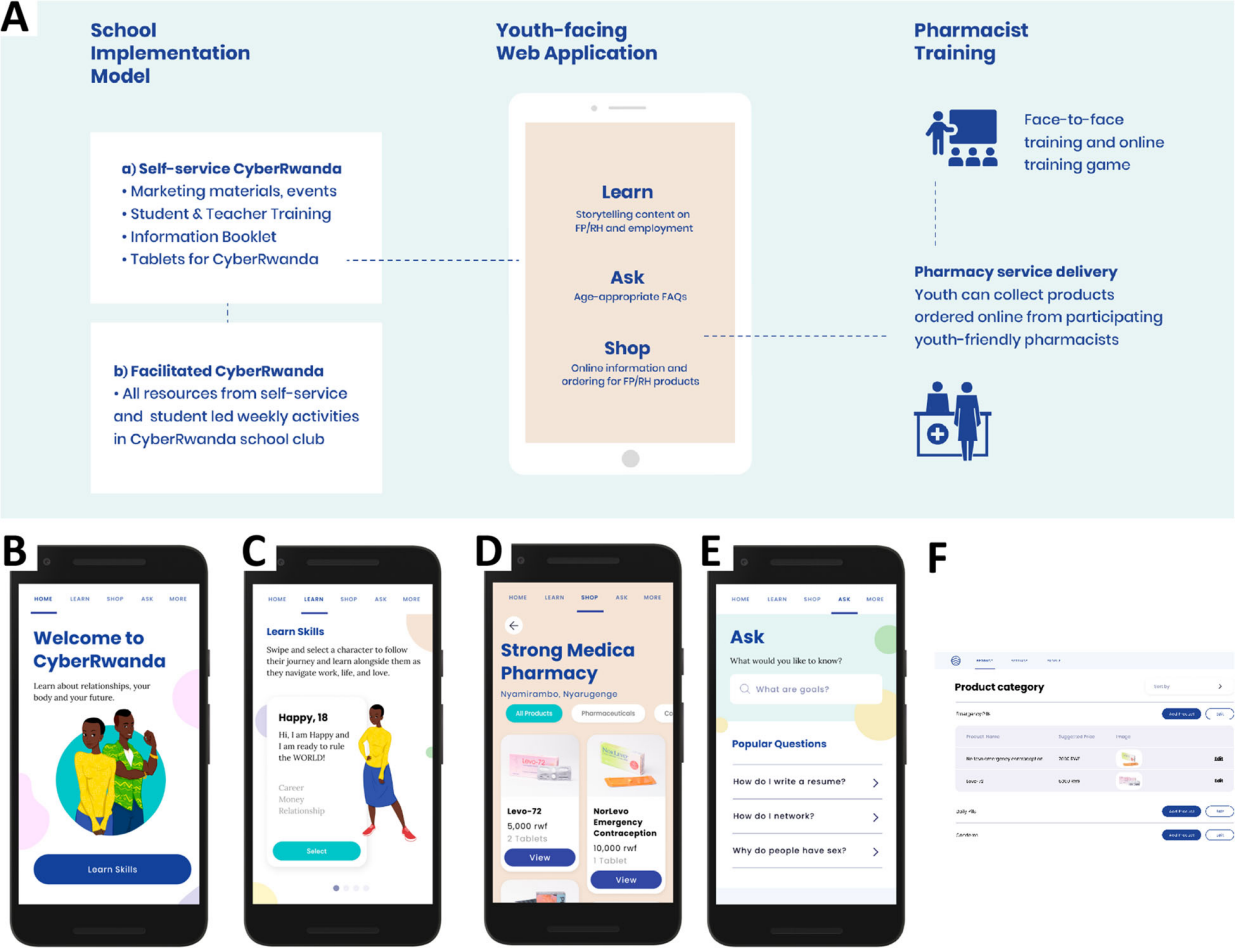
CyberRwanda Intervention Design. **a**: Schematic describing the components of the CyberRwanda digital intervention including the youth-facing web application and pharmacist portal; **b-e**: Images of the youth-facing web application pages [‘Home’ (**b**); ‘Learn’ (**c**); ‘Shop’ (**d**); ‘Ask’ (**e**)]; **f**: Image of the pharmacist portal. Images are the property of YLabs and published with permission of author and YLabs Executive Director Rebecca Hope

The final digital intervention design of CyberRwanda comprised a youth-facing web application^2^ and pharmacist training application. It is delivered through a school implementation model.^3^ A schematic of the intervention is displayed in Fig. 4.

#### Youth-facing web application

There are three main sections of the web application: Learn, Shop and Ask.

##### Learn

Users of CyberRwanda can access edutainment content on FP/RH and economic empowerment. The interactive webcomic follows the two main characters, Happy and Ganza, and their friends, as they navigate adolescence. Characters Mama and Nurse Keza offer advice on pressing health and life questions.

##### Shop

CyberRwanda facilitates the connection between users and local pharmacists who have been trained in providing youth-friendly care. Users can place orders for products such as sanitary pads, pregnancy tests, and contraceptive methods. When required for products such as emergency contraception, users answer medically relevant questions when ordering, and the responses are sent directly to the pharmacist. If they choose, they can enter a phone number to receive order updates via SMS. Online ordering allows youth to avoid uncomfortable interactions with a pharmacist to learn the required information about the products they are purchasing and enables them to collect their order quickly and discreetly from a pharmacist who they know is trained in youth-friendly care.

Young people pay for the products at market rate, with the cost set by the participating pharmacy and viewable on the Cyberwanda online shop. This means that young people are able to more easily ‘shop around’ for the pharmacy with the lowest price. At this stage of the program implementation, vouchers and/or subsidies are not provided for products, though this is a feature that could be feasibly integrated to the platform in future. While young people are able to obtain some products for free or lower cost at health facilities (and can search for these through the CyberRwanda facility finder), our research found that young people prefer to go to pharmacies to purchase products.

##### Ask

Over 200 frequently asked questions are available on the website and searchable by topic, with content written in an age-appropriate and accessible format. Content is aimed towards the lower range of expected users (12–15 years old). Age appropriateness is determined based on the UN International Technical Guidance on Comprehensive Sexuality Education (CSE), upon which the Rwanda Education Board’s CSE curriculum is based. Additionally, all content is validated by Rwanda’s adolescent health and sexual and reproductive health working groups.

#### Pharmacist training application

Pharmacists can elect to join the CyberRwanda network to be listed on the platform as a “youth-friendly” provider and fulfill orders through the site, increasing their potential customer base and revenue. Pharmacists who join the CyberRwanda network must complete training on topics relating to contraceptive methods, national guidelines on providing FP/RH care to adolescents, and how to deliver youth-friendly care. This is delivered through an in-person workshop that counts towards the pharmacists’ continuing professional development credits, as well as a scenario-based online training game.

To help ensure the majority of staff at each participating pharmacy are trained in the CyberRwanda process and providing youth-friendly care, both in-person and virtual training will be provided. Participating pharmacies will be given the option to send either the head pharmacist or nurse to in-person training, and a shorter virtual training will be provided for other staff at the pharmacy. Additionally, to address turnover or the opening of new pharmacies in our target areas, we will conduct refresher trainings.

#### CyberRwanda school implementation model

The CyberRwanda web application is introduced to adolescents in schools through one of two different models, facilitated and self-service, to test whether CyberRwanda requires intensive face-to-face programming in addition to online delivery in order to be effective. The primary difference between the two models is the resource-intensiveness of the implementation. The CyberRwanda self-service model is “lighter-touch,” delivered in schools without peer facilitation and guidance for engaging with the content. A launch event is held at the school to promote the program. Concurrently, training is given to teachers and selected students (“student ambassadors”), along with the provision of tablets and a preloaded internet hotspot, to help students access CyberRwanda during their free time. In contrast, the facilitated version is a more labor-intensive, interactive experience, and potentially effective in response to the insight from prototyping that youth have stronger engagement when learning from and with their peers. In addition to all of the components of the self-service model, the facilitated model also introduces CyberRwanda through school clubs in which trained students (“peer facilitators”) work through an activity booklet and curriculum that accompanies the online material in weekly sessions with their fellow students.

In both the facilitated and self-service implementation models, students interact with CyberRwanda through provided tablets which are connected to the internet. The schools have a degree of discretion over how the tablets are able to be accessed, based on school facilities and programs, supervisory teachers’ capacity, peer facilitators’ preferred modes of engagement, and the school’s preferences. Guidance from the CyberRwanda team mandates a minimum of 5 h/week of access to the tablets, which can be both in unstructured time (e.g. access from the library during lunch hours or after school) and structured time (e.g. classes in which students can access the tablets). Students can also access the tablets via the teachers or from the peer facilitators. Any student in the school is able to access the tablets, not just those who are participating in the study. To overcome the potential barrier of poor internet connectivity in the school, a wifi hotspot with good nation-wide coverage is provided for exclusive use on the CyberRwanda application. A native app is also being developed to allow for future offline use on CyberRwanda tablets, in the case of disruptions in connection.

There is a potential risk of spillover in that students exposed to CyberRwanda may access it on personal phones or tablets outside of the program, and could feasibly share it with friends in non-participating or control schools. Evidence on usage of CyberRwanda from the pilot program suggests that students are unlikely to share CyberRwanda with their friends or attempt to access it outside of school, and the geographical distance between treatment and control schools is intended to minimize the potential for sharing to occur between control and treatment groups.

### Phase 2: implementation and evaluation

Phase 2 includes the implementation and 24-month evaluation of CyberRwanda, which is set to commence in 2020, following in-person school reopening after the COVID-19 crisis.

#### Aims

The study objective is to determine if CyberRwanda can significantly improve health outcomes among youth aged 12–19 as measured through uptake of modern contraceptive methods, delayed initiation of childbearing and increased HIV testing. The secondary objectives are to measure the impact of the CyberRwanda intervention on a set of outcomes related to FP/RH in the following areas: knowledge, behavioral intentions, self-efficacy, social norms and engagement in employment, education, and training.

We are also preparing a cost-effectiveness evaluation to measure the relative cost-effectiveness of the two different implementation models, which will be the subject of a separate protocol.

#### Outcomes

The primary outcomes, measured at the individual participant level, are:

1. Uptake of a modern method of contraception (ages 15–19): The proportion of youth who report currently using a modern method of contraception at endline (24 months)
2. Initiation of childbearing (ages 15–19): The proportion of youth who report having ever been pregnant or fathered a pregnancy at endline (24 months), regardless of the pregnancy outcome
3. HIV testing (ages 12–19): The proportion of youth who have ever had an HIV test at endline (24 months).

While the intervention will be available to 12–19-year-olds at study intervention schools, the first two primary outcomes are measured for 15–19-year-olds for two main reasons: 1) sample size would need to be increased substantially to have sufficient statistical power to detect an effect in the younger age group; and 2) stakeholders were more comfortable focusing the evaluation outcomes on older adolescents to align with social expectations on talking about sexuality. HIV testing is not as stigmatized according to age, because it is routinely conducted irrespective of sexual activity.

Secondary outcomes include the following, measured at the individual participant level for participants aged 15–19 at endline (24 months):

1. Level of knowledge of contraceptive methods and how to prevent pregnancy
2. Behavioral intentions to use contraceptives, pregnancy intentions, intent to discuss contraceptives with partner
3. Self-efficacy related to family planning and reproductive health
4. Social norms related to contraceptive use and other reproductive health behaviors
5. Engagement in employment, education and training: proportion of youth currently in school, training or employment.

#### Study design

The study is a cluster-randomized, non-inferiority trial with secondary schools as the unit of randomization. It is designed as a Hybrid Trial Type 2 Effectiveness-Implementation study [10] to answer two key overarching policy-relevant questions: 1) Do either of the CyberRwanda versions (self-service, facilitated) improve the health and wellbeing of youth compared to schools without access to CyberRwanda? and 2) If one or more of the CyberRwanda versions is found to be effective, is the self-service version at least as effective, within a specified margin, as the facilitated version?

In addition to rigorously measuring the impact of CyberRwanda, we will also include data collection beyond the primary outcomes to learn about acceptability, feasibility, and implementation successes and challenges. This will enable the team at the conclusion of the study to understand not only whether CyberRwanda worked, but why and in what context.

The impact evaluation will use an experimental approach embedded in Proctor’s implementation science framework [15]. Should CyberRwanda be found to be effective, the implementation science framework will allow us to determine potential barriers and facilitators to scale-up and sustainability through input from stakeholders at multiple levels: the individual level (students, peer facilitators, parents, and teachers), the organizational level (schools) and the policy level (community leaders, government officials).

#### Study arms

There are three study arms: 1) CyberRwanda self-service; 2) CyberRwanda facilitated; and 3) control schools, which will receive the standard services that are available in the community. The difference between the self-service and facilitated models is outlined in the section on the CyberRwanda school implementation model, above. Following the end of the evaluation, CyberRwanda will be scaled nationally and made available to control schools.

#### Study population and eligibility criteria

We will recruit 60 schools in 8 districts in Rwanda, randomly selected from a sampling frame of schools in these districts. The study districts are Gatsibo, Nyagatare, Kayonza, Huye, Gasabo, Bugesera, Nyarugenge and Rwamagana. Eligible schools will meet the following criteria:

- Willing to participate (as determined by school leadership)
- Within 4.5 km of a participating pharmacy
- At least 1.5 km from another secondary school to reduce unintended spillover effects
- A minimum of 150 students attending the school
- Day schools only (no boarding or mixed schools).

Within participating schools, irrespective of the study arm, we will recruit all students enrolled in levels S1 and S2 (secondary school grades 1 and 2) at baseline to enroll in the evaluation cohort. Late entry to school and repetition of levels leads to age variation within levels in Rwanda [16]. Data from the pilot study showed this range of levels will yield the most students within the target age group. Eligibility criteria for students is as follows:

- Attending a study school at baseline
- Between 12 and 19 years of age
- Consent (for participants ages 18–19) or assent (for participants under 18) provided to participate
- Parental consent provided (for participants under 18)
- Willingness to provide valid contact information for study follow-up purposes.

#### Randomization

Randomization will occur at the cluster (school) level, stratified by district. There is no concealment at the cluster level, but individual participants in the control schools will not be explicitly told about the intervention. The study is not blinded.

Society for Family Health Rwanda (SFH) is responsible for recruiting schools into the study pool and inviting them to join a district meeting. The YLabs team will support a participatory randomization process at the district meeting. All schools that agree to participate will move forward to the randomization process (data on the proportion of schools who are approached and who subsequently refuse participation will be recorded). At each district meeting, we will ask each school representative to choose a ball out of a bag for their district. Color coded balls will represent which arm of the study the school will be randomly allocated: control, CyberRwanda self-service, or CyberRwanda facilitated. Participants will not see the color of the ball that they draw from the bag, nor will they know the significance of the color until all the balls have been drawn.

The head teacher at the participating schools provides consent for their school to be included within the study. This is agreed before knowing which arm (control or either treatment) the school will receive. Consent is provided by participating students if over 18 years old. Students under 18 provide assent and consent from their parents. Parental consent forms will be distributed at school to all students in S1 and S2 in study schools. Students will be instructed to bring the consent forms home to parents/guardians, ask them to read and sign them, and bring them back to school within the next week. Study staff will return 1 week after distributing consent forms to collect signed parental consent forms for students, and record parental consent status for each student. Request for withdrawal from the study by any individual participant or school is respected and enabled. Model consent forms can be found in Additional file 4.

#### Sample size calculations

The sample size was determined for the primary outcome of uptake of a modern contraceptive method. Power and sample size analyses were conducted for the primary analysis using 2000 simulated datasets (created in R statistical software) constructed based on historic data on the proportion of sexually active youth currently using a modern contraceptive method.

The primary analysis is powered at 80% to detect the presence of an intervention effect when CyberRwanda facilitated has a relative risk of at least 2.5. Although this relative risk seems large, in absolute terms, the baseline percentage of girls using contraception is only 2%, so this would mean increasing that to 5%. To achieve this power, we need to enroll 60 schools and 50 girls per school (equal cluster size is assumed). When we include boys (who are not part of the primary outcome on which the study is powered), this means we will enroll a total of 100 students per school, for a total of 6000 students in the study.

#### SPIRIT diagram

**Fig. 5:**
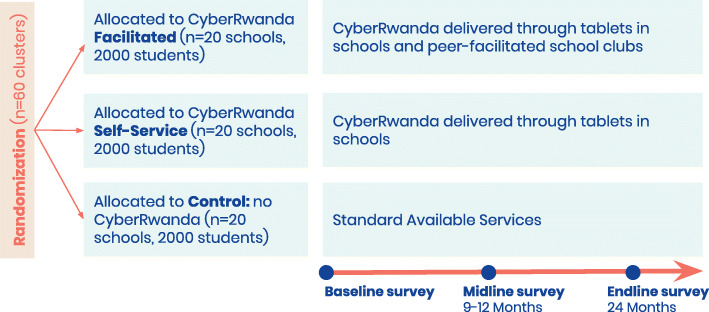
Cluster Randomized Hybrid Effectiveness-Implementation Trial Study Design. Schematic of the study design

The SPIRIT flow diagram for this trial is included in Fig. 5, and completed checklist included in Additional file 3.

#### Data collection

Study participants will be asked to complete three surveys at three different time points during the study: one at baseline, one at midline (9–12 months after launch), and one at the end of the study (after 24 months). Surveys will be administered in Kinyarwanda using a tablet computer and Qualtrics offline survey software and will take 30–45 min to complete. The survey will cover the following broad topic areas: sociodemographic characteristics, knowledge about FP/RH, social norms related to sexual health, gender roles, norms related to gender-based violence, intentions related to having a family, use of contraceptives, communication with partners, sexual behavior, childbearing, future aspirations and goal-setting, and attitudes and intentions toward school and employment (see draft baseline questionnaire in Additional file 2).

In addition to the surveys, continuous, real-time data will be collected using Google Analytics to understand user engagement patterns (e.g. drop-off points in character storylines, online ordering behaviors) in all three arms.

As preparation for implementation, we conducted an eight-week pilot study in September to October 2019 in 4 schools and 2 youth centers in Nyagatare and Kigali (Nyarugenge and Gasabo districts), to refine the implementation model and data collection instruments.

Data will be strictly managed and stored securely to maintain participant confidentiality and minimize the risk of potential harms such as accidental disclosure. Study Investigators as specified in the IRB documents will have access to the final study dataset. Full details of data management and monitoring protocols can be found in Additional file 5.

#### Student follow-up

The impact evaluation carries a high risk of attrition as we are enrolling a cohort of students at baseline and will assess outcomes 24 months later. We can expect that over the 2 years of the impact study between 5 and 20% of students will drop out of school, and girls are more likely to be out of school than boys from age 15 and up [16]. Efforts to retain members of each cohort – even if they leave school – will be crucial given that reasons for dropping out of school are closely related to our primary outcomes (i.e. childbearing), and any significant differential attrition between study arms will bias results. We will mitigate the risk of study dropout by collecting multiple modes of contact for each enrolled student and parents at baseline, as well as providing participants small communication payments (<$1 USD) for regularly updating contact information every school term so we can retain updated contact information. In addition to these efforts, we have also built in to our power calculations that we can expect a 10% attrition rate over the course of the study.

#### Data analysis

To first assess the presence of an intervention effect, we will utilize a generalized linear mixed model (GLMM) with uptake of a modern contraceptive method (binary outcome) and initiation of childbearing (binary outcome) as the outcome variables. The model will include a binary explanatory variable for the intervention group (control vs. CyberRwanda in either the self-service or facilitated arm) with control group as the reference, and model terms for the stratification factor (district) as well as other relevant variables measured at baseline (e.g. age, sex). We will present relative risks and 95% confidence intervals using robust standard errors to account for clustering at the school and district levels.

If there is evidence of a statistically significant intervention effect relative to control, we will proceed with the assessment of non-inferiority to understand whether CyberRwanda self-service is non-inferior to CyberRwanda facilitated within a specified margin. To do this, the coefficients on the categorical variable for the intervention groups can be used as approximations of the log relative risks (RRs) comparing the probability of contraceptive uptake (or initiation of childbearing) in the intervention arms compared to control arm. We will use a one-sided Wald-style test of non-inferiority, based on an a priori specified non-inferiority margin of 50% of the lower bound of the confidence interval for CyberRwanda facilitated.

#### Implementation science framework

As a Hybrid Trial Type 2 Effectiveness-Implementation study, the design of this study is rooted in implementation science. We will evaluate not only if CyberRwanda is effective in achieving its intended health outcomes, but also which of two different school-based implementation models is most effective. In addition, we are considering all of the relevant contextual aspects to get a deeper understanding of why the different programmatic versions were or were not effective, using the constructs from Proctor’s implementation science framework. We are interested in understanding barriers and facilitators of use of the CyberRwanda platform among the youth, as well as implementation at participating schools and the perceptions and experiences of school staff, the parents, the pharmacists, and community leaders. In each of these groups we will explore the Proctor implementation science constructs: acceptability, adoption, appropriateness, cost, feasibility, fidelity, penetration, and sustainability, through semi-structured interviews conducted during the impact evaluation, and through short surveys embedded into the CyberRwanda platform. If one or more of the CyberRwanda versions is found through this analysis to be effective, logical questions follow about adapting the intervention for scale that are traditionally beyond the scope of narrow impact evaluations. In the scenario whereby neither CyberRwanda version has impact on the primary outcomes, the implementation indicators can shed light on where there are gaps in the program impact pathway.

## Discussion

This study represents one of the few rigorously-evaluated digital health interventions for youth at a large scale in a low- or middle-income country. While digital health interventions are becoming increasingly popular, there is a paucity of evidence on their effectiveness in facilitating adolescent health behavior change in a low- or middle-income setting [17]. Furthermore, most digital health evaluations measure change in knowledge as a result of intervention [18]; this evaluation extends the impact pathway to also measure behavioral outcomes (uptake of modern contraceptive methods and HIV testing) and impact (initiation of childbearing). By structuring the evaluation as a Hybrid Trial Type 2 Effectiveness-Implementation research design, this study will contribute not only to an understanding of whether CyberRwanda itself is effective at increasing adolescent contraceptive use and HIV testing, but whether a school-based implementation model that complements online content with peer-facilitated face-to-face activities is more effective than purely online delivery.

The human-centered design methodology used to develop CyberRwanda is an important means of increasing the likelihood of implementation success. User-centered strategies have the potential to increase both the impact and sustainability of efforts to promote uptake of evidence-based practices, by improving the fit between those practices and their implementation contexts [19]. Recent meta-analyses have found that the majority of adolescent FP/RH interventions in Sub-Saharan Africa have only led to moderate behavior change at best [20–23], with the common critique that programs show limited understanding of the specific vulnerabilities and experiences of youth in their social/cultural context, or failure to overcome implementation challenges [24]. Given this context, a human-centered design approach that centers the needs, barriers, and behavioral drivers of youth at the core of the intervention offers a promising path forward.

Another distinguishing feature of CyberRwanda is that it supports adolescents throughout the whole journey from learning to accessing care. Most digital health interventions focus on providing information or directing users to care. To our knowledge, there are no existing integrated digital education tools that target both youth and healthcare providers with information, facilitate guided referrals to public health facilities, and provide online ordering of health products. CyberRwanda aims to both increase adolescents’ demand for modern contraceptive methods through its educational components and to streamline supply by offering discreet and confidential product ordering and equipping pharmacists to deliver youth-friendly care. This holistic, systems approach aims to overcome some of the barriers that persist for adolescents trying to access appropriate FP/RH care.

There are a number of limitations and challenges to this research. We outline the three that appear most salient.

First, the primary outcomes that the evaluation measures are self-reported. Responses may be subject to social desirability bias if respondents misreport answers to sensitive questions regarding sexual behavior, use of contraceptives, and pregnancy history. In an effort to mitigate misreporting, the informed consent process will reassure participants that their answers are entirely confidential, and interviews will take place using best practices in a private space where respondents feel comfortable. A number of different procedures for conducting the interviews were tested during the pilot study to identify the optimal approach.

Second, the results of the impact study will be generalizable only to students, not to the broader population of youth who have dropped out of school or never attended school.

Third, there is a risk of contamination of students in our control group who may have exposure to CyberRwanda if they hear about it from others and access it outside of school. During the pilot study, very few students accessed CyberRwanda outside school and on other devices, so even if control students hear about CyberRwanda, we do not have strong evidence that contamination will be a significant problem. However, we will monitor for this possibility during the study. Additionally, an inclusion criterion for schools in the study is that they are at least 1.5 km from another participating school, to minimize risk of spillover. In addition to this, we are restricting publicity of the CyberRwanda site to only school-level promotion; we will not have any official presence on social media, nor will we use any other efforts to promote the site beyond the individual schools. There may be spillover from other ongoing projects addressing health and well-being of adolescents in the regions we are working, but given we are randomizing schools within districts, we would hope any exposure to other community programs would occur with similar frequency in both intervention and control schools. At the midline and endline surveys, we will also ask participants in the control schools if they have ever heard of CyberRwanda, if so, where they heard about it, and if they have ever used it, and if so, what aspects of the site they have engaged with. We will also ask all participants if they have participated in other programs addressing sexual and reproductive health of adolescents.

This study highlights some of the considerations involved in rigorously evaluating a digital health intervention. One major appeal of a digital intervention is that it can easily reach a wide audience through mass promotion, including through social/mainstream media and personal referrals. However, for the evaluation to have high internal validity, access to the intervention must be tightly controlled to prevent contamination of the control group; a tradeoff the team weighed against the benefits of the possibility for localized ‘viral’ growth.

The evaluation of CyberRwanda will not only build an understanding of the model’s effectiveness and inform decisions for scale but will also add considerable value to the field by growing the evidence base on the design and implementation of digital health interventions in low resource settings.

## Supplementary Information

**Additional file 1.** Prototypes Tested. List of the major prototypes tested throughout Phase 1 (Intervention Design), and the key hypotheses and insights gleaned.

**Additional file 2.** Draft Questionnaire. Draft questionnaire for baseline data collection.

**Additional file 3.** SPIRIT checklist. Completed SPIRIT checklist.

**Additional file 4.** Model Consent Forms. Model consent form and other related documentation given to participants and authorized surrogates.

**Additional file 5.** Data Management and Monitoring Protocols. Detailed methods on data management, monitoring, and dissemination, in compliance with SPIRIT guidelines.

## Data Availability

The datasets used and/or analyzed during the current study are available from the corresponding author on reasonable request. This includes datasets used in sample size/power calculations and from the pilot study.

## Abbreviations

CSE: Comprehensive Sexuality Education
DHS: Demographic and Health Survey
FP/RH: Family Planning and Reproductive Health
HIV: Human Immunodeficiency Virus
LMIC: Low or Middle Income Country
RCT: Randomized Controlled Trial
RR: Relative Risk
SFH: Society for Family Health
SMS: Short Message Service
STI: Sexually Transmitted Infection
TPB: Theory of Planned Behavior

## Footnotes

1 In many cases, participants were fluent in English, and conducting sessions in English did not require translation. However, where Kinyarwanda translation was required, the translator was trained in the subject matter of the sessions to enable clearer translations and a smoother flow of the conversation.

2 A native app is being developed in addition to the web application to overcome barriers of poor internet coverage in some regions. The structure and content of the native app is the same as the web app, so we have focused our discussion on the web app for simplicity.

3 In addition to a school implementation model, CyberRwanda is also being delivered in one Youth Center in each study district in order to meet the needs of out-of-school youth. Implementation in Youth Centers is not part of the experimental study and is therefore not a focus of this paper.
