# Supplementary material for: Design and impact evaluation of a digital reproductive health program in Rwanda using a cluster randomized design: study protocol
Source: BMC Public Health. 2020 Nov 13;20:1701. doi: 10.1186/s12889-020-09746-7 (PMC7662730; doi:10.1186/s12889-020-09746-7)
Supplement: Supplementary file 1 — Additional file 1. Prototypes Tested. List of the major prototypes tested throughout Phase 1 (Intervention Design), and the key hypotheses and insights gleaned. [file 12889_2020_9746_MOESM1_ESM.docx]

**Prototypes Tested**

The following is a list of the major prototypes tested throughout Phase 1, and the key hypotheses and insights gleaned.

**Pocket Guide to Success** | Target: Youth

- **Description:** Small booklet that covers key messages on FP/RH and job readiness through stories of youth characters preparing for their future
- **Hypothesis:** Youth will be engaged and learn if FP/RH and employment skills content is integrated into storytelling about a girl’s journey to achieving her goals.
- **Insights:** Results from a pre-post survey illustrated the efficacy of the content in The Pocket Guide to Success in improving attitudes and knowledge about FP/RH.

**Ask Juliette** | Target: Youth

- **Description:** Virtual nurse who answers questions and offers referrals to a local youth corner in a clinic.
- **Hypothesis:** Youth will be more comfortable asking questions to a health provider through a confidential online platform.
- **Insights:** Youth are more comfortable seeking sensitive FP/RH information online than in person, but a virtual nurse does not increase their self-reported likelihood of visiting a clinic.

**Avatars** | Target: Youth

- **Description:** Youth-designed self-representing avatars to interact with the app based content
- **Hypothesis:** Youth will be more engaged if characters on the app look like them, and are designed by them.
- **Insights:** Youth enjoy designing avatars and appreciate that characters look like them, but don’t want to represent themselves in the app because that removes a layer of anonymity.

**Videos** | Target: Youth

- **Description:** Videos produced by youth designers surrounding the topics of professionalism, job skills, and youth access to FP/RH services and clinics
- **Hypothesis:** Youth will be engaged and learn from video content, especially if it is produced by other young people.
- **Insights:**  Youth want a peer voice, not a teacher or educator, so youth-designed videos resonate well.

**Menstrual journey maps and hormonal diagrams** | Target: Youth

- **Description:** Tests of different styles of age-appropriate communication about menstruation, hormones and fertility
- **Hypothesis:** Certain framing of messages around menstruation and puberty will reduce fear and myths and promote understanding.
- **Insights:** ‘Gateway’ topics such as puberty and protection from HIV make content about FP/RH more accessible and culturally acceptable.

**Baza Mutoni Textline** | Target: Youth

- **Description:** A textline tool that participants could use to ask questions anonymously
- **Hypothesis:** Given the importance of confidentiality and a taboo around openly discussing FP/RH, youth will feel more comfortable asking questions to a textline than to someone in-person.
- **Insights:** Youth want a peer voice, not a teacher or educator. Confidential answers to questions from a trusted source are very desirable.

**Boy’s Rulebook** | Target: Youth

- **Description:** A ‘rulebook’ of content for boys which was developed to communicate the role of boys in reducing unintended teen pregnancy, as well as develop attitudes about consent and knowledge about consent laws
- **Hypothesis:** Boys are often the drivers of sexual decision-making of girls, but rarely receive adequate education on FP/RH, so a tool that explains FP/RH from a boy’s perspective will develop knowledge around key topics such as contraception and consent.
- **Insights:** Boys appreciate FP/RH content that is framed from a boy’s perspective, and are responsive to discussions about consent.

**Motorcycle Contraceptive Delivery** | Target: Youth

- **Description:** A motorcycle delivery service for girls to purchase and acquire contraceptives
- **Hypothesis:** Motorcycle delivery might reduce some barriers to purchasing contraception.
- **Insights:** Youth find home delivery too risky in terms of confidentiality, and would prefer to pick up products from a retailer.

**Youth Navigator** | Target: Providers

- **Description:** Youth counseling tool that offers guidance on how to communicate about all contraceptive methods without bias
- **Hypothesis:** When providers do not offer youth-friendly care, it is because of a lack of knowledge or awareness, rather than a deliberate choice. Providing training will be sufficient to improve pharmacists’ quality of FP/RH care to youth.
- **Insights:** Pharmacists crave more training and instruction, but a more in-depth training may be required than a simple counselling tool.

**Youth Expert Network** | Target: Providers

- **Description:** A WhatsApp chat group among providers to discuss technical questions, learn from medical experts in the FP/RH network, and share challenges and learnings with other FP/RH providers
- **Hypothesis:** A youth-focused professional chat forum will help address misconceptions and fill-in youth-relevant knowledge gaps that providers have (target bias due to lack of knowledge).
- **Insights:** Providers are not always willing to use their personal cell phones for work purposes, so WhatsApp may not be the best medium for sharing professional development lessons.

**Youth Bill of Rights** | Target: Providers

- **Description:** A visual resource to communicate service quality standards so that young people and providers alike know what good service looks like
- **Hypothesis:** ​Knowing what quality youth-friendly services looks like will change provider behavior with their youth patients and vice versa, so youth are more equipped to advocate for better care.
- **Insights:** A youth bill of rights is not enough to overcome the reluctance for youth to access services in a clinic due to the lack of privacy.

**Social Permissions** | Target: Providers

- **Description:** A series of testimonials from key influencers which document their support for contraception and their positive experience using it
- **Hypothesis:** Testimonials from key stakeholders will make providers feel like they can “break the taboo” and provide hormonal contraception to unmarried youth.
- **Insights:** Testimonials are not enough to overcome the main barrier to youth service uptake at clinics: the risk of a confidentiality breach.

**Express Feedback** | Target: Providers

- **Description:** A post-consultation SMS, digital, or print survey for patients to rapidly report on quality of care
- **Hypothesis:** Offering clients a way to provide feedback will incentivize providers to change their behavior towards clients.
- **Insights:** Clients found the process took too long, but the opportunity for confidential feedback was desirable.

**Contraceptive Trivia** | Target: Providers

- **Description:** An interactive game designed to assess provider knowledge and myths around different contraceptive methods
- **Hypothesis:** A non-threatening, interactive game will encourage providers to consider what myths and inaccuracies they may hold regarding different contraceptive methods.
- **Insights:** Providers enjoy the gamified way of learning but need more in-depth training to answer all of their questions and dispel myths around contraception.

**Contraceptive Cheat Sheets** | Target: Providers

- **Description:**​ Rapid, visual information for nurses and pharmacists to help them offer correct information when counseling youth on contraceptives
- **Hypothesis:** A Kinyarwanda cheat sheet will address knowledge gaps and misinformation of providers, enabling them to offer the right contraceptive for the youth seeking their advice.
- **Insights:** Pharmacists did not often refer to the tool during counselling, but some displayed it for their clients’ benefit.

**Express Pass** | Target: Providers & Youth

- **Description:** A completed paper or SMS intake form that youth present to a pharmacist to avoid personal (but medically necessary) questions, thereby ensuring client privacy in a busy space
- **Hypothesis:** By answering sensitive questions on paper, youth might be more inclined to be honest about their reproductive needs, and pharmacists will be able to save time and feel confident they are providing the correct product.
- **Insights:** ​This prototype was met with great enthusiasm amongst providers and youth. Youth appreciated the opportunity to place an order for contraceptive methods and respond to medically necessary questions via SMS, rather than providing that information in-person. Providers felt that this service could be complementary to their existing services and appreciate the confidentiality of the service.

**Contraceptive Bundle** | Target: Providers & Youth

- **Description:** A CyberRwanda-branded bundle of emergency contraception, condoms and the oral contraceptive pill for sale
- **Hypothesis:** Given youth predominantly ask pharmacists for emergency contraception despite the potential for other forms of contraception to be more suitable, this bundle might introduce youth to more appropriate contraception options.
- **Insights:** ​Pharmacists expressed concern about having emergency contraception and the oral contraceptive pills in the same package, believing that access to many pills at once could result in misuse or overdose.

**Wake-up Call** | Target: Providers & Youth

- **Description:** A free service allowing youth to receive an SMS reminder to take their oral contraceptive pill or return for follow-up injections
- **Hypothesis:** Providers crave more oversight with youth to ease their conscience and ensure medication is taken correctly. This tool will allow pharmacists more oversight of youth follow-up and encourage them to provide contraceptive products to youth more readily.
- **Insights:** ​Providers (both pharmacists and nurses) responded very positively to this prototype and reported that they would be more comfortable prescribing oral contraceptive pills to youth clients if this service existed. Given the prominent stigma regarding oral contraceptive pills among unmarried women, however, it was challenging to find young women who would admit to using the pill and who would be willing to participate in this service during prototyping.

**Chutes and Ladders** | Target: Providers

- **Description:** A gamified training module on youth-friendly FP/RH care provision that dramatizes the impact of providers’ bias on a young person’s contraception options and future
- **Hypothesis:** Providers will be engaged and learn through this game and as a result be more likely to change their practices to become more youth-friendly.
- **Insights:** ​Most providers who played the game said it can teach providers about youth-friendly care and are interested in playing multiple times. There was interest from the Rwandan Pharmacy Association to develop the game into a broader training module and use the tool in a facilitated group training setting.

**Contraceptive Palm Card** | Target: Providers & Youth

- **Description:** A CyberRwanda-branded palm card that pharmacists can give youth clients outlining directions for oral contraceptive pill and emergency contraception use
- **Hypothesis:** Given pharmacists generally do not trust youth clients to take oral contraception correctly, this guide will allow pharmacists to have peace of mind that youth are receiving the information they need, and give youth the information they require to be safe in an easy-to-understand and engaging format.
- **Insights:** ​Both providers and youth responded positively to the prototype and some of them requested to keep the prototypes. Youth were interested in sharing with their friends, and felt safe about keeping the palm cards with them.

**Refer-a-friend** | Target: Providers & Youth

- **Description: ​**A physical card that pharmacists can give to youth, which incentivizes youth to refer their friends to pharmacists who provide high quality service
- **Hypothesis:** Having financial and social incentives through client referrals will improve the quality of provider service.
- **Insights:** ​We found it is rare for youth to discuss private information related to sexual and reproductive health. As a result, this prototype was abandoned during the early stages of prototyping since its success was dependent on youth word-of-mouth.
