# Supplementary material for: Design and impact evaluation of a digital reproductive health program in Rwanda using a cluster randomized design: study protocol
Source: BMC Public Health. 2020 Nov 13;20:1701. doi: 10.1186/s12889-020-09746-7 (PMC7662730; doi:10.1186/s12889-020-09746-7)
Supplement: Supplementary file 2 — Additional file 2. Draft Questionnaire. Draft questionnaire for baseline data collection. [file 12889_2020_9746_MOESM2_ESM.docx]

CyberRwanda - Pilot Survey

Start of Block: Screener and Informed Consent

Q1 **Recruitment, Screener and Informed Consent**

Q2 Data collector ID

________________________________________________________________

| 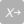 |
| --- |

Q3 District

▼ Gasabo (1) ... Nyagatare (2)

| 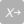 |
| --- |

Q4 Name of School

▼ Nyagatare Secondary School (1) ... Groupe Scolaire Kabuga (3)

Q5 SID (Enter from Potential Participant Form)

________________________________________________________________

| 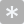 |
| --- |

Q6 Re-enter SID (From Participant Form)

________________________________________________________________

| 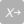 |
| --- |

Q7
**[READ TEXT ALOUD]**


 Hello, my name is _____________. I am a public health researcher. I am conducting research on a new intervention called CyberRwanda that was delivered to your school through Society for Family Health and YLabs, a non-profit working with adolescents in Rwanda. Participation in this research includes taking a survey. It should take about 45 minutes to complete the survey.   Do you have any questions?   Would you like to participate?”

- Yes (1)
- No (2)

Skip To: End of Survey If [READ TEXT ALOUD] Hello, my name is _____________. I am a public health researcher. I am conducti... = No

| 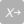 |
| --- |

Q8 Before we begin, we want to make sure you qualify for our study. Please indicate your age:

- Under 12 years of age (1)
- Above 19 years of age (2)
- 12-17 years of age (3)
- 18-19 years of age (4)

Skip To: End of Survey If Before we begin, we want to make sure you qualify for our study. Please indicate your age: = Under 12 years of age

Skip To: End of Survey If Before we begin, we want to make sure you qualify for our study. Please indicate your age: = Above 19 years of age

| 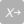 |
| --- |

Q9
[READ ALOUD]

Which grade are you studying in?

- S1 (1)
- S2 (2)
- S3 (3)
- S4 (4)
- Any other grade (5)

Skip To: End of Survey If [READ ALOUD] Which grade are you studying in?  = Any other grade

Display This Question:

If Before we begin, we want to make sure you qualify for our study. Please indicate your age: = 12-17 years of age

| 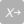 |
| --- |

Q10 **[DO NOT READ]**
 
RA: Has parent/guardian of participant provided signed consent?

- Yes (1)
- No (2)

Skip To: End of Survey If [DO NOT READ]   RA: Has parent/guardian of participant provided signed consent? = No

Q11 If potential participant is eligible, say, “Thank you for your interest in participating in our study. If you would like we can sit down and talk more about the study and I can tell you about the benefits and risks. You can then ask me questions and think about whether you would like to be in the study.” (Proceed to informed assent/consent)    If potential participant is not eligible, let them know they are not eligible and thank them for their time.

Display This Question:

If Before we begin, we want to make sure you qualify for our study. Please indicate your age: = 12-17 years of age

| 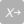 |
| --- |

Q12

INFORMED ASSENT
IntroductionMy name is ___________________ and I am working for _______at YLabs and _________ at Society for Family Health in Kigali and ______ at YLabs based in Kigali and Berkeley, California. SFH and YLabs are organizations working together to improve the health of young people in Rwanda. I am also working with Professor Sandra McCoy at the University of California, Berkeley and Dr. Aline Umubyeyi at the University of Rwanda to conduct a research study. We invite you to participate in this study. What is a research study?A research study is when people like me collect a lot of information about a certain thing to find out more about it. Before you decide if you want to be in this study, it’s important for you to understand why we’re doing the research and what’s involved. Please read this form carefully. You can discuss it with your parents or anyone else. If you have questions about this research, just ask me. Why are we doing this study?We are doing this study is to understand health and wellbeing among students in schools located in this district of Rwanda. This study is not part of your school work, and you won't get grades on it. Why are we talking to you about this study?We’re inviting you to take part because you go to a school where we are doing the study, are in level S1 through S4, and belong to the age group we are interested in studying. What will happen if you are in this study?
If you agree to be in this study, and your parents do not say no, we will ask you to:• Respond to a questionnaire
We will ask you to respond to a questionnaire that will be administered by a research assistant who is part of our study staff. The research assistant will ask you a set of questions and then fill out your responses on a computer tablet. The questionnaire will take about 45 minutes. Each questionnaire is generally about your health and well-being and includes some questions on sensitive topics including sexual behavior and health history, and work-related questions about employment and wages.• Be interviewedSome study participants will also be invited to an interview to talk about your experience being part of the study, including what you liked and didn’t like about it and what could be improved. The interview will last about 60 minutes during a mutually agreed upon time. With your permission, we will audiotape and take notes during the interview. Study Location and Time: We will do the survey at a private place at your school. It will take about 45 minutes of your time. We will do the interview at a private place also at your school, at a time that works for you. The interview will take about 60 minutes. The total amount of time required for you to participate in the study is one hour and 45 minutes. Are there any benefits to being in the study?There is no benefit to you personally for taking part in this study, but we hope that the results of the research will help improve ways of reaching youth/students with information on their health and well-being in the future and use of digital tools such as tablets to reach adolescents with information on sexual and reproductive health. Are there any risks or discomforts to being in the study?You might get bored or tired and decide that you don’t want to finish the survey. If so, just tell us that you want to stop.You may feel uncomfortable with some of the questions we ask you. If so, you can ask to skip a question, or you can feel free to stop at any time.If you get very upset during the questionnaire, a school counselor or a nurse from SFH will be available for you to talk to.You might feel uncomfortable being audio recorded. If so, we can turn off the recorder at any time. A possible risk for any research is that people outside the study might get hold of confidential study information. We will do everything we can to make sure that doesn't happen. 

Who will know about your study participation?Only the researchers will know the details of your study participation. If we publish reports or give talks about this research, we will only discuss group results. We will NOT use your name or any other personal information that would identify you. To help protect confidentiality, we will give your study data a code number, and protect the data in a file with a password that only the researchers know. The file will be on a computer that only the researchers are allowed to use. Any paper forms that contain your name and other personal identifying information will be stored in a locked cabinet in a locked office in a secured building. After the study is complete, we plan to keep only data that has all identifiable information removed for 10 years, in case we or other researchers want to use the study information for other studies. Will you get paid for being in the study?You will not be paid for being in this study. Do you have to be in the study?No, you don’t. Research is something you do only if you want to. No one will get mad at you if you don’t want to be in the study. And whether you decide to participate or not, either way will have no effect on your grades at school. And remember, you can always change your mind later if you don't want to be in the study any more. Do you have any questions?You can contact us if you have questions about the study, or if you decide you don’t want to be in the study any more. You can talk to me, or your parents, or someone else at any time during the study if you like. You can contact Janepher Turatsinze from SFH at 0788309996 or email:jturatsinze@sfhrwanda.org, or you can contact Dr. Sandra McCoy in the United States at +1- 510-642-0513 or smccoy@berkeley.edu. If you have any questions or concerns about your child’s rights and treatment as a research subject, you may contact the Rwanda National Ethics Board at +250 255107884 or the office of the University of California, Berkeley's Committee for the Protection of Human Subjects, at +1-510-642-7461 or subjects@berkeley.edu 
****************************************** ASSENT OF ADOLESCENT (12–17 years old)If you decide to participate, and your parents provide consent for you to participate we'll give you a copy of this form to keep for future reference. If you would like to be in this research study, please sign your name on the line below.________________________________________   _______________ Child's Name/Signature (printed or written by child)* Date________________________________________   _______________ Signature of Investigator/Person Obtaining Assent Date******************************************  If verbal assent only is being obtained: Investigator or Person Conducting Assent Discussion: Initial here if child cannot sign, to document that child received this information and gave assent verbally: ______
 
**DID PARTICIPANT SIGN OR VERBALLY ASSENT TO PARTICIPATE?**

- Yes (1)
- No (2)

Skip To: End of Survey If INFORMED ASSENT Introduction My name is ___________________ and I am working for _______at YLabs... = No

Display This Question:

If Before we begin, we want to make sure you qualify for our study. Please indicate your age: = 18-19 years of age

| 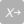 |
| --- |

Q13 **INFORMED CONSENT
Introduction**   My name is ___________________ and I am working for __________ at Society for Family Health in Kigali and ______ at YLabs based in Kigali and Berkeley, California. SFH and YLabs are organizations working together to improve the health of young people in Rwanda. I am also working with Professor Sandra McCoy at the University of California, Berkeley and Dr. Aline Umubyeyi at the University of Rwanda to conduct a research study. We invite you to participate in this study.   Before you decide whether to be part of this study, it’s important for you to understand why we’re doing the research and what’s involved.  Please read this form carefully. If you have any questions about the research, feel free to ask us.   **Purpose** We are doing this study to understand more about the health and well-being of students between the ages of 12 and 19 who are enrolled in secondary schools in levels S1 through S4 across several districts in Rwanda. We are inviting you to participate because you are in this age range, attend one of the study schools, and are currently enrolled in level S1 through S4 at one of the study schools. The school has agreed that we can conduct the research here. This study is not part of your school work and it will not be graded.   Procedures If you agree to be in this study we will ask you to:   ·      Respond to a questionnaire We will ask you to respond to a questionnaire that will be administered by a research assistant who is part of our study staff. The research assistant will ask you a set of questions and then fill out your responses on a computer tablet.  The questionnaire will take about 30-45 minutes. The survey is generally about your health and well-being and includes some questions on sensitive topics including sexual behavior and health history, and work-related questions about employment and wages.   ·      Be interviewed Some study participants will also be invited to an interview to talk about your experience being part of the study, including what you liked and didn’t like about it and what could be improved. The interview will last about 60 minutes during a mutually agreed upon time. With your permission, we will audiotape and take notes during the interview.   **Study Location and Time:**  We will do the survey and the interview at a private place at your school. The survey will take about 30-45 minutes of your time and the interview will take about 60 minutes, so the total time if you are interviewed will be 1 hour and 45 minutes.   **Benefits** There is no benefit to you personally for taking part in this study.  However, we hope that the results of the research will help improve ways of reaching youth/students with information on their health and well-being in the future.   Risks/Discomforts   ·      You may get bored or tired and decide that you do not want to complete the study activities. If so, you can just tell us that you wish to stop.   ·      You may feel uncomfortable with some of the questions we ask you. If so, you can ask to skip a question, or you can feel free to stop at any time.   ·      If you get very upset during the questionnaire, a school counselor or a nurse from SFH will be available for you to talk to.   ·      *Breach of Confidentiality*: A possible risk for any research is that confidentiality could be compromised, that is, people outside the study might get hold of confidential study information.  We will do everything we can to minimize this risk.   **Confidentiality** We will keep your study data as confidential as possible.  If we publish or present results of this study, we will not use individual names or other personally identifiable information.  All the data will be handled as confidentially as possible. To protect confidentiality, we will do the following: ·      Any study information in the study database will not include your name and contact information. All of your information will instead be identified with a random code. ·      Your research records will always be securely stored. Paper forms that contain personal identifying information will be stored in a locked cabinet in a locked office in a secured building. Computer databases that contain your data will be stored in an encrypted format on password-protected computers and will require a password so that only authorized study personnel have access. ·      Publications or presentations with results of this study will not use individual names or any other personally identifiable information.   Future use of study data Identifiers might be removed from the identifiable private information. After such removal, the information could be used for future research studies or distributed to other investigators for future research studies without additional informed consent from the subject or the legally authorized representative.   **Compensation/Payment** You will not be paid for being in this study.   Rights   ***Participation in research is completely voluntary****.*  Research is something you do only if you want to. No one will get mad at you if you don’t want to be in the study. And whether you decide to participate or not, either way will have no effect on your grades at school. And remember, you can always change your mind later if you don't want to be in the study any more.   **Do you have any questions?** You can contact us if you have questions about the study, or if you decide you don’t want to be in the study any more. You can talk to me, or your parents, or someone else at any time during the study if you like.   You can contact Janepher Turatsinze from SFH at **0788309996** or email**:** jturatsinze@sfhrwanda.org Or you can contact Dr. Sandra McCoy in the United States at +1-510-642-0513 or smccoy@berkeley.edu.   If you have any questions or concerns about your child’s rights and treatment as a research subject, you may contact the Rwanda National Ethics Board at  +250 255107884 or the office of the University of California, Berkeley's Committee for the Protection of Human Subjects, at +1-510-642-7461 or subjects@berkeley.edu   ****************************************** **CONSENT OF ADOLESCENT (18 years old or above)**   If you decide to participate in this study, ***please sign and date below***. We will give you a copy of this form to keep for future reference.   ___________________________________ Participant Name (*please print*)                                                                                                   _______________ Participant Signature                                                              Date                                                                                                   _______________ Signature of Investigator/Person Obtaining Consent              Date  **DID PARTICIPANT SIGN INFORMED CONSENT FORM?**

- Yes (1)
- No (2)

Skip To: End of Survey If INFORMED CONSENT Introduction   My name is ___________________ and I am working for __________ at... = No

End of Block: Screener and Informed Consent

Start of Block: 1. Demographic Characteristics

Q14 [READ TEXT ALOUD]

Thank you for talking with me today. Today, we are going to complete a survey for the study you are enrolled in. I will be asking you questions about you, your household, and your everyday life. We will also talk about your relationships, your knowledge of health and your current sexual and reproductive health. Your responses will remain entirely confidential, so please keep this in mind as you answer these questions as honestly as possible. If you have any questions or if anything I ask is unclear, please stop me and I will do my best to give you a better explanation. Please remember this survey has nothing to do with your classes or homework or grades. Nothing you tell me here will affect any of your school performance.  Let’s get started with the survey. We will begin by asking you a little about yourself and your family.

Q15 **Section 1: Demographic Characteristics**

| 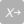 |
| --- |

Q16 **[DO NOT READ]**

 Sex of the respondent

- Male (1)
- Female (2)

| 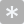 |
| --- |

Q17 How old were you at your last birthday?

________________________________________________________________

| 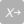 |
| --- |

Q18
What is the current grade/class you are at?

- S1 (1)
- S2 (2)
- S3 (3)
- S4 (4)

| 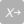 |
| --- |

Q19 What is your current marital status?

- No Partner (1)
- Partnered, Boyfriend or Girlfriend (2)
- Married (3)
- Other, specify (4) ________________________________________________

Display This Question:

If What is your current marital status? = Partnered, Boyfriend or Girlfriend

Or What is your current marital status? = Married

| 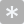 |
| --- |

Q20 How old is your partner?

________________________________________________________________

| 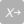 |
| --- |

Q21 What is your current living arrangement?
[READ OPTIONS ALOUD]

- Living with partner/wife/husband (1)
- Living with both parents (2)
- Living with father only (3)
- Living with mother only (4)
- Living with a guardian/relative (5)
- Living alone (6)
- Boarding school (7)
- Other, specify (8) ________________________________________________

Display This Question:

If What is your current living arrangement? [READ OPTIONS ALOUD] != Boarding school

Q22 What is the main mode of transportation you currently use to travel from home to school?

- Walk (1)
- Bicycle (2)
- Motorbike (6)
- Bus (3)
- Car (family driver) (4)
- Other, specify (5) ________________________________________________

Display This Question:

If What is the main mode of transportation you currently use to travel from home to school? = Bus

And What is your current living arrangement? [READ OPTIONS ALOUD] != Boarding school

Q23 How much does it cost to ride the bus from home to the school?

________________________________________________________________

Display This Question:

If What is your current living arrangement? [READ OPTIONS ALOUD] != Boarding school

| 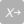 |
| --- |

Q24 How long does the total journey from home to school usually take?

- Less than 5 mins (1)
- 5-10 mins (2)
- 10-15 mins (3)
- 15-20 mins (4)
- More than 20 mins (5)

| 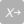 |
| --- |

Q25 What is the main mode of transportation you would use to travel from school to the pharmacy?

- Walk (1)
- Bicycle (2)
- Motorbike (6)
- Bus (3)
- Car (family driver) (4)
- Other, specify (5) ________________________________________________

Display This Question:

If What is the main mode of transportation you would use to travel from school to the pharmacy? = Bus

Q26 How much does it cost to ride the bus from school to the pharmacy?

________________________________________________________________

| 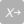 |
| --- |

Q27 How long would it take the total journey from school to the pharmacy?

- Less than 5 mins (1)
- 5-10 mins (2)
- 10-15 mins (3)
- 15-20 mins (4)
- More than 20 mins (5)

Display This Question:

If What is your current living arrangement? [READ OPTIONS ALOUD] != Boarding school

| 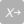 |
| --- |

Q28 What is the main mode of transportation you would use to travel from the pharmacy to home?

- Walk (1)
- Bicycle (2)
- Motorbike (6)
- Bus (3)
- Car (family driver) (4) (4)
- Other (5): ___________________ (5) ________________________________________________

Display This Question:

If What is the main mode of transportation you would use to travel from the pharmacy to home? = Bus

And What is your current living arrangement? [READ OPTIONS ALOUD] != Boarding school

Q29 How much does it cost to ride the bus from the pharmacy to home?

________________________________________________________________

Display This Question:

If What is your current living arrangement? [READ OPTIONS ALOUD] != Boarding school

| 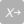 |
| --- |

Q30 How long would it take the total journey (by all modes) from the pharmacy to home?

- Less than 5 mins (1)
- 5-10 mins (2)
- 10-15 mins (3)
- 15-20 mins (4)
- More than 20 mins (5)

| 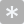 |
| --- |

Q31 How many people live in your household and regularly (at least 4 times a week) eat meals together? [Please include yourself]

________________________________________________________________

| 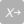 |
| --- |

Q32
What language do you primarily speak at home?

- Kinyarwanda (1)
- French (2)
- Swahili (3)
- English (4)
- Other, specify (5) ________________________________________________

| 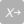 |
| --- |

Q33 What is your religion?

- Catholic (1)
- Protestant (2)
- Adventist (3)
- Muslim (4)
- Jehovah’s Witness (5)
- Traditional (6)
- No religion (7)
- Refuse to answer (8)
- Other, specify (9) ________________________________________________

| 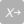 |
| --- |

Q34 Is your father alive?

- Yes (1)
- No (2)

| 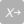 |
| --- |

Q35 What is / was his level of education?

- None (1)
- Some primary (2)
- Completed primary (3)
- Some secondary (4)
- Complete secondary (5)
- Vocational training (6)
- Completed some university (7)
- Completed university (8)
- Don’t know (9)

| 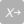 |
| --- |

Q36 What is / was his major source of income?

- Farming / agriculture (1)
- Police officer / armed forces (2)
- Shop or hotel worker (3)
- Shop or hotel owner (4)
- Buying and selling items (5)
- Manual labor (portering, offloading trucks, etc.) (6)
- Trade (construction, tailoring, etc.) (7)
- Profession (teacher, healthcare) (8)
- Government job (9)
- Other, specify (10) ________________________________________________
- None / Unemployed (11)
- Don’t know (12)

| 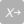 |
| --- |

Q37 Is your mother alive?

- Yes (1)
- No (2)

| 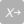 |
| --- |

Q38 What is / was her level of education?

- None (1)
- Some primary (2)
- Completed primary (3)
- Some secondary (4)
- Complete secondary (5)
- Vocational training (6)
- Completed some university (7)
- Completed university (8)
- Don’t know (9)

| 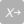 |
| --- |

Q39 What is / was her major source of income?

- Housewife (1)
- Farming / agriculture (2)
- Police officer / armed forces (3)
- Shop or hotel worker (4)
- Shop or hotel owner (5)
- Buying and selling items (6)
- Manual labor (portering, offloading trucks, etc.) (7)
- Trade (construction, tailoring, etc.) (8)
- Profession (teacher, healthcare) (9)
- Government job (10)
- Other, specify (11) ________________________________________________
- None / Unemployed (12)
- Don’t know (13)

Q40 How many brothers and sisters did/do you have in total, including those who do not live with you and those who have died?    **[NOTE: If none, write 0]**

- Brothers living (1) ________________________________________________
- Brothers dead (4) ________________________________________________
- Sisters living (5) ________________________________________________
- Sisters dead (29) ________________________________________________

| 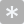 |
| --- |

Q41 How many of your living brothers and sisters are older than you?    **[NOTE: If none, write 0]**

________________________________________________________________

| 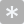 |
| --- |

Q42 How many people live in your home? By home we mean those people in your family who live together and regularly eat from the same pot.

________________________________________________________________

Q43 Now I will ask you a few questions about any activities that generate income for you, your family or your household.

| 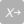 |
| --- |

Q44 Do you currently have a small business or income-generating activity (IGA) that you either started yourself or run by yourself?

- Yes (1)
- No (2)

Display This Question:

If Do you currently have a small business or income-generating activity (IGA) that you either starte... = Yes

| 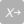 |
| --- |

Q45 Please select every kind of business or IGA you started or run yourself

- Farming / agriculture / animal care or rearing (1)
- Making / selling arts and crafts (2)
- Education / tutoring (3)
- Shop owner (4)
- Casual / physical work (5)
- Food vending (6)
- Charcoal burning (7)
- Brick making (8)
- Skilled trade (construction, tailoring, carpentry, weaving, etc.) (9)
- Fishing (10)
- Domestic household chores (11)
- Buying and reselling products (12)
- Other, specify (13) ________________________________________________

Display This Question:

If Do you currently have a small business or income-generating activity (IGA) that you either starte... = Yes

Q46 In what month and year did you start your (first) business?

- Month (1) ________________________________________________
- Year (2) ________________________________________________

Display This Question:

If Do you currently have a small business or income-generating activity (IGA) that you either starte... = Yes

Q47 Counting backwards from today, how much money did you earn from the business / businesses in the past 2 months?

________________________________________________________________

Display This Question:

If Do you currently have a small business or income-generating activity (IGA) that you either starte... = Yes

| 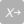 |
| --- |

Q48 What do you do with the money you earn from the business/businesses? Please select the answer that says where most of your money goes.

- Reinvest in my business (1)
- Reinvest in another business (2)
- Save for a future project or goal (3)
- Pay school fees (4)
- Buy school supplies (5)
- Pay for daily expenses like food (6)
- Give to my family (7)
- Leisure (8)
- Other, specify (9) ________________________________________________

| 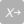 |
| --- |

Q49 Are you paid to work at someone else’s business or income-generating activity?

- Yes (1)
- No (2)

Display This Question:

If Are you paid to work at someone else’s business or income-generating activity?  = Yes

| 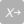 |
| --- |

Q50 Please select every kind of work that you do for someone else.

- Police officer / soldier (1)
- Shop or hotel worker (2)
- Stone quarrying (3)
- Manual labor (portering, offloading trucks, etc.) (4)
- Food vending (5)
- Charcoal burning (6)
- Brick making (7)
- Farming / agriculture / animal care or rearing (8)
- Skilled trade (construction, tailoring, carpentry, weaving, etc.) (9)
- Fishing (10)
- Domestic household chores (11)
- Other, specify (12) ________________________________________________

Display This Question:

If Are you paid to work at someone else’s business or income-generating activity?  = Yes

| 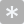 |
| --- |

Q51 How much money did you earn from this activity/ these activities in the past 2 months?

________________________________________________________________

Display This Question:

If Are you paid to work at someone else’s business or income-generating activity?  = Yes

| 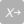 |
| --- |

Q52 Did you receive anything other than money for any work in the past 2 months?

- Yes, specify (1) ________________________________________________
- No (2)

Display This Question:

If Are you paid to work at someone else’s business or income-generating activity?  = Yes

| 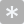 |
| --- |

Q53 If you did receive something other than money, how much do you think is the total value of these items?

________________________________________________________________

| 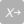 |
| --- |

Q54 Do you usually contribute with your time to household activities such as farming, agriculture, animal rearing, or working at the family business without receiving a wage?

- Yes (1)
- No (2)

Display This Question:

If Do you usually contribute with your time to household activities such as farming, agriculture, an... = Yes

| 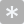 |
| --- |

Q55 In the past week, how many hours did you spend working in household activities?

________________________________________________________________

Display This Question:

If Do you usually contribute with your time to household activities such as farming, agriculture, an... = Yes

| 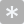 |
| --- |

Q56 If you were to be paid for this work, how much do you think you should be paid per week?

________________________________________________________________

End of Block: 1. Demographic Characteristics

Start of Block: 2. Exposure to Media, Sources of Information

Q57 **Section 2: Exposure to Media, Sources of Information**

Q58 **[READ TEXT ALOUD]** In this section, I will ask you about your exposure to media including TV, radio, mobile phones and social media.  The following questions are about your access to and use of media.

Q59 **[READ TEXT ALOUD]**

 For each item that I mention, please tell me whether you have access to it and what kind of access you have. By access we mean, you may not own it, but you can use it if you need.

Q60 Where do you access the following list of items? (Please select only one option per item)

|  | Through my own device (1) | Through a borrowed device (2) | At the Youth Center (3) | Do NOT have access (4) |
| --- | --- | --- | --- | --- |
| Computer, Laptop, iPad/Tablet (1) |  |  |  |  |
| Internet (4) |  |  |  |  |
| Feature phone [NOTE: NOT a smart phone] (2) |  |  |  |  |
| Smart Phone (3) |  |  |  |  |

| 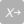 |
| --- |

Q61 Do you have any social media account, such as Facebook, WhatsApp, Twitter, etc?    [SELECT ALL THAT APPLY]

- Facebook (1)
- Twitter (2)
- WhatsApp (3)
- Instagram (4)
- SnapChat (5)
- No, I do not have any social media accounts (6)
- Other, specify (7) ________________________________________________

Q62 **[READ TEXT ALOUD]**

 The next few questions are about how you seek information on sexual and reproductive health. Some of these questions can be embarrassing, but remember that everything you tell me is private.

| 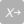 |
| --- |

Q63 Young people learn about puberty and sexual and reproductive systems of men and women - I mean the ways in which boys' and girls' bodies change during the teenage years - from many sources.  They may learn from teachers at school, parents, brothers and sisters, from friends, from doctors or they may learn from books, films and magazines.  
 What/who has been the most important source of information for you on this topic?

- Teacher (1)
- Mother (2)
- Father (3)
- Brother (4)
- Sister (5)
- Other family members (6)
- Friends/peers (7)
- Youth Center staff (8)
- Doctors (9)
- Books/magazine (10)
- Films/videos (11)
- Internet/social media (12)
- Other, specify (13) ________________________________________________

| 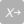 |
| --- |

Q64 From whom, or where, would you prefer to have received more information on this topic?

- Teacher (1)
- Mother (2)
- Father (3)
- Brother (4)
- Sister (5)
- Other family members (6)
- Friends/peers (7)
- Youth Center staff (8)
- Doctors (9)
- Books/magazines (10)
- Films/Videos (11)
- Internet/Social media (12)
- Other, specify (13) ________________________________________________

| 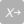 |
| --- |

Q65 Some schools have classes on puberty, on sexual, reproductive systems, relationships between boys and girls.  Has your school ever organized any classes for you on these topics?

- Yes (1)
- No (2)
- Dont know (3)

Skip To: End of Block If Some schools have classes on puberty, on sexual, reproductive systems, relationships between boys... = No

Skip To: End of Block If Some schools have classes on puberty, on sexual, reproductive systems, relationships between boys... = Dont know

Display This Question:

If Some schools have classes on puberty, on sexual, reproductive systems, relationships between boys... = Yes

| 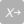 |
| --- |

Q66 Have you ever attended school classes on any of these topics?
  Please note this or any other question has nothing to do with your grades in school.

- Yes (1)
- No (2)
- Dont Know/ Dont Remember (3)

Skip To: End of Block If Have you ever attended school classes on any of these topics?   Please note this or any other que... = No

Skip To: End of Block If Have you ever attended school classes on any of these topics?   Please note this or any other que... = Dont Know/ Dont Remember

Display This Question:

If Have you ever attended school classes on any of these topics?   Please note this or any other que... = Yes

| 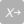 |
| --- |

Q67 How useful did you find these classes? 
[READ OPTIONS ALOUD]

- Useful (1)
- Somewhat useful (2)
- Not useful (3)
- Cannot say (4)

End of Block: 2. Exposure to Media, Sources of Information

Start of Block: 3. Knowledge of Fertility, Contraception and HIV

Q68 **Section 3: Knowledge of Fertility, Contraception and HIV**

Q69 **[READ TEXT ALOUD]**   You’re doing well so far. This section will ask you questions about your knowledge of fertility, contraception – ways of avoiding getting pregnant - and HIV.   Now I have some questions on fertility awareness and reproduction.

| 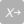 |
| --- |

Q70
From one menstrual period to the next, are there certain days when a woman is more likely to become pregnant?

- Yes (1)
- No (2)
- Don't know (3)

Skip To: Q72 If From one menstrual period to the next, are there certain days when a woman is more likely to beco... = No

Skip To: Q72 If From one menstrual period to the next, are there certain days when a woman is more likely to beco... = Don't know

| 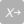 |
| --- |

Q71
Is this time just before her period begins, during her period, right after her period has ended, or halfway between two periods? 


[READ OPTIONS ALOUD]

- Just before her period begins (1)
- During her period (2)
- Right after her period has begun (3)
- Half-way between two periods (4)
- Other (5)
- Don't know (6)

| 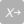 |
| --- |

Q72 What is the sign that a girl is now able to become pregnant?

- First menstruation / First bleeding (1)
- Don't know (2)
- Other (3)

Q73
 **[READ TEXT ALOUD]**
 
For this question and the next question I will read you some statements. Please tell me whether you think the statement is true or false.

| 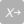 |
| --- |

Q74
Using some methods of contraception, such as the pill, IUD or the depo shot, will make me unable to have children in the future.

- True (1)
- False (2)
- Don't know (3)

| 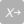 |
| --- |

Q75 Emergency contraception can be taken up to one month after having unprotected sex to prevent a pregnancy.

- True (1)
- False (2)
- Don't Know (3)

| 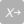 |
| --- |

Q76 Now I would like to talk about contraception - I mean ways in which men and women can avoid getting pregnant.  I am going to read a list of methods. Which methods have you heard of?     **[NOTE: READ OPTIONS AND DESCRIPTION ALOUD]**

|  | Yes (1) | No (2) |
| --- | --- | --- |
| The PILL: Women can take a pill every day to avoid becoming pregnant (1) |  |  |
| The IUD: Women can have a T-shaped piece of plastic that contains copper or hormones (2) |  |  |
| FEMALE STERILIZATION: Women can have an operation to avoid having any more children. (5) |  |  |
| MALE STERILIZATION: Men can have an operation to avoid having any more children. (7) |  |  |
| INJECTABLES: Women can have an injection by a health provider that stops them from becoming pregnant for one or more months. (9) |  |  |
| IMPLANTS: Women can have one or more small rods placed in their upper arm by a doctor or nurse which can prevent pregnancy for one or more years (10) |  |  |
| CONDOMS: Men can put a rubber sheath on their penis before sexual intercourse. (11) |  |  |
| FEMALE CONDOMS: Women can place a sheath in their vagina before sexual intercourse. (12) |  |  |
| EMERGENCY CONTRACEPTION: As an emergency measure, within three days after they have unprotected sexual intercourse, women can take special pills to prevent pregnancy. (15) |  |  |
| LACTATIONAL AMENORRHEA METHOD (LAM): LAM is a form of natural birth control that relies on the new mother feeding her baby only breastmilk for up to six months and having no periods or spotting during that time. (16) |  |  |
| STANDARD DAYS METHODS (SDM)/ CALENDAR METHOD: The woman know days of the month when she can get pregnant by using beads, a smartphone app or a calendar (17) |  |  |
| RHYTHM METHOD: Every month that a woman is sexually active she can avoid pregnancy by not having sexual intercourse on the days of the month she is most likely to get pregnant. (18) |  |  |
| WITHDRAWAL: Men can be careful and pull out before climax (19) |  |  |
| Any other ways or methods that women or men can use to avoid pregnancy? (20) |  |  |

Display This Question:

If [DO NOT READ] Sex of the respondent  = Female

| 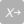 |
| --- |

Q77
For girls and women there are tools that you can use to track your menstrual cycle so that you know when your fertile period (when you are most likely to get pregnant) is. Can you name some of these tools?
 
**[SELECT ALL THAT APPLY]**

- Beads (1)
- Calendar (2)
- Thermometer (3)
- Smartphone Apps (4)
- Other, specify (5) ________________________________________________
- Don't know any tools (6)

| 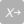 |
| --- |

Q78
Are you currently doing anything to avoid pregnancy?

- Yes (1)
- No (2)
- Refuse to answer (3)
- Don’t know (4)

Display This Question:

If Are you currently doing anything to avoid pregnancy? = Yes

| 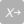 |
| --- |

Q79 What are you and your partner using to avoid pregnancy?


[READ OPTIONS ALOUD. SELECT ALL THAT APPLY]

- Pills (1)
- Condoms (2)
- Female Condoms (3)
- IUD / Intrauterine devices (4)
- Injectables (5)
- Implants/ Jadelle (6)
- Emergency Contraceptive Pills (7)
- Diaphragm (8)
- Foam/Jelly (9)
- Standard Days Method (10)
- Female Sterilization (11)
- Male Sterilization (12)
- Lactational Amenorrhea Method (LAM) (13)
- Periodic Abstinence (14)
- Withdrawal (15)
- Nothing (16)
- Misoprostol (17)
- Other (Specify) (18) ________________________________________________
- Don’t know (19)
- Refuse to answer (20)

Display This Question:

If [DO NOT READ] Sex of the respondent  = Female

| 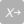 |
| --- |

Q80 Have you ever used emergency contraception?

- Yes (1)
- No (2)
- Don’t know (3)
- Refuse to answer (4)

Display This Question:

If [DO NOT READ] Sex of the respondent  = Female

| 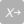 |
| --- |

Q81 Have you ever been pregnant, regardless of the outcome of the pregnancy or if no child was born?  

- Yes (1)
- No (2)
- Don’t know (3)
- Refuse to answer (4)

Q82 [READ TEXT ALOUD]
Now I would like to talk about HIV/AIDS and ask you a few knowledge questions related to HIV/AIDS.

| 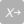 |
| --- |

Q83 Can the risk of HIV/AIDS virus transmission be reduced by having sex with only one uninfected partner who has no other partners?

- Yes (1)
- No (2)
- Don't know (3)

| 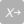 |
| --- |

Q84 Can a person reduce the risk of getting HIV/AIDS virus by using a condom every time they have sex?

- Yes (1)
- No (2)
- Don't know (3)

| 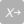 |
| --- |

Q85 Can a healthy-looking person have HIV/AIDS virus?

- Yes (1)
- No (2)
- Don't know (3)

| 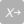 |
| --- |

Q86 Can people get the HIV/AIDS virus because of witchcraft or other supernatural means?

- Yes (1)
- No (2)
- Don't know (3)

| 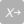 |
| --- |

Q87 Can a person get HIV/AIDS virus by sharing food with someone who is infected?

- Yes (1)
- No (2)
- Don't know (3)

| 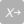 |
| --- |

Q88 Can people get the HIV/AIDS virus from mosquito bites?

- Yes (1)
- No (2)
- Don't know (3)

| 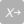 |
| --- |

Q89 Can men reduce their risk of getting the HIV/AIDS virus by getting circumcised?

- Yes (1)
- No (2)
- Don't know (3)

End of Block: 3. Knowledge of Fertility, Contraception and HIV

Start of Block: 4A. Sexual Behavior (FEMALES)

Q90 **Section 4A: Sexual Behavior (FEMALES)**

Q91
How old were you the first time you had vaginal sex/sexual intercourse?

- 12 years old or younger (5)
- 13 years (7)
- 14 years (8)
- 15 years (9)
- 16 years (10)
- 17 years (11)
- 18 years (12)
- 19 years (13)
- Never had sex (14)

Skip To: End of Block If How old were you the first time you had vaginal sex/sexual intercourse?   = Never had sex

| 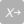 |
| --- |

Q92 The first time you had sexual intercourse with someone would you say you were willing, somewhat willing, or not willing at all to? Willing means you gave permission or said it was OK, or that you did it because you wanted to and not because someone made you.
[READ OPTIONS ALOUD]

- Very willing (1)
- Somewhat willing (2)
- Not willing at all (3)
- Refuse to answer (4)

| 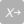 |
| --- |

Q93 Have you ever had sex in exchange for money, food, gifts, drugs, alcohol, shelter or other goods?

- Yes (1)
- No (2)
- Don’t know (3)
- Refuse to answer (4)

Q94 [READ TEXT ALOUD]
Now I’m going to ask you to think about your current partner, or if you don’t currently have a partner, your most recent partner.

| 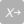 |
| --- |

Q95 When was the last time you had sexual intercourse with this partner?


[READ OPTIONS ALOUD]

- Within the last 6 months (1)
- 6-12 months ago (2)
- A year or more ago (3)

| 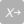 |
| --- |

Q96 Did you ever discuss using contraception with this partner?

- Yes (1)
- No (2)
- Refuse to answer (3)

| 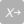 |
| --- |

Q97 The last time you had sex with this partner, did you do anything to avoid pregnancy?

- Yes (1)
- No (2)
- Don’t Know (3)
- Refuse (4)

Display This Question:

If The last time you had sex with this partner, did you do anything to avoid pregnancy? = Yes

| 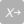 |
| --- |

Q98 What did you use?
 
[READ OPTIONS ALOUD. SELECT ALL THAT APPLY]

- Pills (1)
- Condoms (2)
- Female Condoms (3)
- IUD / Intrauterine devices (4)
- Injectables (5)
- Implants/ Jadelle (6)
- Emergency Contraceptive Pills (7)
- Diaphragm (8)
- Foam/Jelly (9)
- Standard Days Method (10)
- Female Sterilization (11)
- Male Sterilization (12)
- Lactational Amenorrhea Method (LAM) (13)
- Periodic Abstinence (14)
- Withdrawal (15)
- Nothing (16)
- Misoprostol (17)
- Other, specify (18) ________________________________________________
- Don’t know (19)
- Refuse to answer (20)

Display This Question:

If The last time you had sex with this partner, did you do anything to avoid pregnancy? = Yes

| 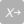 |
| --- |

Q99 Where did you or your partner get this method?     [READ OPTIONS ALOUD]   **[NOTE: Probe to identify the type of source. If unable to determine if public or prvate sector, write the name of the place]**

- Referral hospital (1)
- Provincial/District hospital (2)
- Health Center (3)
- Health Post (4)
- Outreach (5)
- Community Health Worker (6)
- Other Public Health Facility (7) ________________________________________________
- Polyclinic (8)
- Clinic (9)
- Dispensary (10)
- Pharmacy (11)
- Family planning clinic (12)
- Other private medical sector (13)
- Kiosk/Shop/Bar (14)
- Church (15)
- Friend/Relative (16)
- Youth Center (17)
- Other, specify (18) ________________________________________________

Display This Question:

If The last time you had sex with this partner, did you do anything to avoid pregnancy? = No

Or The last time you had sex with this partner, did you do anything to avoid pregnancy? = Don’t Know

Or The last time you had sex with this partner, did you do anything to avoid pregnancy? = Refuse

| 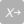 |
| --- |

Q100 Young people have different reasons for not using contraception. Tell me which reasons best describe why you and your partner are not currently using contraception, or why you didn’t use contraception with your most recent partner. 

 [DO NOT READ OPTIONS. SELECT ALL THAT APPLY]   [NOTE: **If no current partner, insert “why didn’t you use contraception with your last partner”]**

- I am too embarrassed to talk about using contraception (1)
- I want to get pregnant (2)
- It is too hard to get my partner to use contraception with me (3)
- Contraception interferes with enjoyment (4)
- I don’t know where to get contraception (5)
- I don’t want to seem too eager for sex (6)
- I don’t think I could get pregnant (7)
- I have never really thought about it (8)
- I can’t afford it (9)
- I’m embarrassed to buy it (10)
- People will judge me for using it/buying it (11)
- Other (12)
- Refuse to answer (13)

| 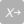 |
| --- |

Q101
Now still thinking about the last time you had sex with this partner, were you aware if your partner used anything to delay or avoid pregnancy?

- Yes (1)
- No (2)
- Don't know (3)
- Refuse (4)

| Page Break |  |
| --- | --- |

Q102 Reproductive Autonomy Scale   **[READ TEXT ALOUD]**   The next questions are about you and your main partner or a recent sexual partner. The questions ask about who has the most say in different types of decisions. Having the “most say” means if there was a disagreement, the person who would have final say. If you have more than one partner, think about your main partner. If you don’t have a partner, think about a previous partner. If you have not had to make any of the following decisions, please think about who would have the most say in the decision.

| 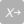 |
| --- |

Q103
**[READ TEXT ALOUD]**
  For these questions, please select one of the following response choices:
  My sexual partner (or someone else such as a parent or mother in-law/father in-law)  Both me and my sexual partner (or someone else such as a parent or mother in-law /father in-law) equally  Me

|  | My partner (1) | My partner & me (2) | Me (3) |
| --- | --- | --- | --- |
| Who has the MOST say about whether you use a method to prevent pregnancy? (1) |  |  |  |
| Who has the MOST say about which method you would use to prevent pregnancy? (4) |  |  |  |
| Who has the MOST say about when you should have a baby in your life? (5) |  |  |  |
| If you or your partner became pregnant but it was unplanned, who would have the MOST say about whether you would raise the child, seek adoptive parents, or have an abortion? (6) |  |  |  |

| 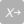 |
| --- |

Q104 **[READ TEXT ALOUD]** The next questions are about you and your main or most recent sexual partner. For these questions, please select one of the following response choices:
 Strongly Disagree Disagree  Agree Strongly Agree

|  | Strongly Disagree (1) | Disagree (2) | Agree (3) | Strongly Agree (4) |
| --- | --- | --- | --- | --- |
| My partner has stopped me from using a method to prevent pregnancy when I wanted to use one (1) |  |  |  |  |
| My partner has messed with or made it difficult to use a method to prevent pregnancy when I wanted to use one (4) |  |  |  |  |
| My partner has made me use a method to prevent pregnancy when I did not want to use one (8) |  |  |  |  |
| If I wanted to use a method to prevent pregnancy my partner would stop me (5) |  |  |  |  |
| My partner has pressured me to become pregnant (6) |  |  |  |  |

End of Block: 4A. Sexual Behavior (FEMALES)

Start of Block: 5. Pregnancy History

Q105 **Section 5: Pregnancy History**

Q106 **[READ TEXT ALOUD]**   Next I am going to ask you some questions about pregnancy and children. Your responses will remain entirely confidential, so please keep this in mind as you answer these questions as honestly as possible. If you have any questions or if anything I ask is unclear, please stop me and I will do my best to give you a better explanation. Please remember this survey has nothing to do with your classes or homework or grades. Nothing you tell me here will affect any of your school performance.

| 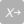 |
| --- |

Q107 I want to make certain that I have the correct information. When asked if you had ever had sexual intercourse in your life, you said

- Yes (1)
- No (2)
- Refuse to Answer (3)

Skip To: End of Block If I want to make certain that I have the correct information. When asked if you had ever had sexual... != Yes

Display This Question:

If I want to make certain that I have the correct information. When asked if you had ever had sexual... = Yes

Or Have you ever had sexual intercourse, VAGINAL sex? By this, we mean when a man or boy put’s his p... = Yes

| 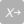 |
| --- |

Q108 Have you ever been pregnant by anyone? Even if you have had an abortion or miscarriage in the past.

- Yes (1)
- No (2)
- Refuse to Answer (3)

Skip To: End of Block If Have you ever been pregnant by anyone? Even if you have had an abortion or miscarriage in the past. != Yes

| 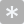 |
| --- |

Q109 How old were you when you became pregnant for the first time?

________________________________________________________________

| 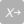 |
| --- |

Q110 Have you ever given birth to a child?

- Yes (1)
- No (2)

Skip To: Q112 If Have you ever given birth to a child? = No

Display This Question:

If Have you ever given birth to a child? = Yes

| 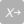 |
| --- |

Q111 Including babies that did not survive, how many times have you given birth?    **[NOTE: If none, record ‘00’]**

- Sons (1) ________________________________________________
- Daughters (2) ________________________________________________

| 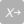 |
| --- |

Q112 Are you currently pregnant?

- Yes (1)
- No (2)

Display This Question:

If Are you currently pregnant? = Yes

| 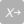 |
| --- |

Q113 Just before you became  pregnant, would you say that you

- Intended to get pregnant (1)
- Your intentions kept changing (2)
- You did not intend to get pregnant (3)

End of Block: 5. Pregnancy History

Start of Block: 6. FPRH Service Utilization

Q114 **Section 6: FPRH Service Utilization**

Q115 **[READ TEXT ALOUD]**


 Now we are going to talk about your health and your body, and whether you have been to a clinic and accessed services. We will ask you specifically about HIV testing, STI treatments and pregnancy testing.

| 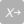 |
| --- |

Q116 I don’t want to know the results, but have you ever been tested for HIV/AIDS?

- Yes (1)
- No (2)

Display This Question:

If I don’t want to know the results, but have you ever been tested for HIV/AIDS? = Yes

| 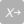 |
| --- |

Q117 How many months ago was your most recent HIV test?
 
**[NOTE: If more than 24 months, write in number of years]**

- Months (1) ________________________________________________
- Years (2) ________________________________________________

| 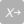 |
| --- |

Q118 Have you ever used an HIV self-test (an HIV test you can buy at the store and use on yourself at home)?

- Yes (1)
- No (2)

Display This Question:

If If How old were you at your last birthday?      Text Response Is Greater Than or Equal to 15

And And How old were you at your last birthday?      Text Response Is Less Than or Equal to 19

And I want to make certain that I have the correct information. When asked if you had ever had sexual... = Yes

| 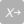 |
| --- |

Q119 Have you received family planning counseling services in the past 12 months?

- Yes (1)
- No (2)

Display This Question:

If Have you received family planning counseling services in the past 12 months? = Yes

| 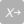 |
| --- |

Q120 Where did you receive FP counseling services?

- Referral hospital (1)
- Provincial/District hospital (2)
- Health Center (3)
- Health Post (4)
- Outreach (5)
- Community Health Worker (6)
- Other Public Health Facility (7)
- Polyclinic (8)
- Clinic (9)
- Dispensary (10)
- Pharmacy (11)
- Family planning clinic (12)
- Other private medical sector (13)
- Kiosk/Shop/Bar (14)
- Church (15)
- Friend/Relative (16)
- Youth Center (17)
- Other, specify (18) ________________________________________________

| 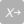 |
| --- |

Q121 During the last 12 months, have you been told by a health care provider that you have an infection which you got through sexual contact?

- Yes (1)
- No (2)
- Don't know (3)

Display This Question:

If During the last 12 months, have you been told by a health care provider that you have an infectio... = Yes

| 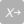 |
| --- |

Q122 Did you get the infection treated by a medical provider like a doctor, nurse?

- Yes (1)
- No (2)
- Don't know (3)

Display This Question:

If Did you get the infection treated by a medical provider like a doctor, nurse? = Yes

| 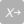 |
| --- |

Q123  Where did you get it treated?

- Referral hospital (1)
- Provincial/District hospital (2)
- Health Center (3)
- Health Post (4)
- Outreach (5)
- Community Health Worker (6)
- Other Public Health Facility (7)
- Polyclinic (8)
- Clinic (9)
- Dispensary (10)
- Pharmacy (11)
- Family planning clinic (12)
- Other private medical sector (13)
- Kiosk/Shop/Bar (14)
- Church (15)
- Friend/Relative (16)
- Youth Center (17)
- Other, specify (18) ________________________________________________

Display This Question:

If [DO NOT READ] Sex of the respondent  = Female

| 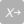 |
| --- |

Q124 In the last 12 months, have you taken a pregnancy test?

- Yes (1)
- No (2)
- Don't Know (3)
- Refuse to Answer (4)

Display This Question:

If In the last 12 months, have you taken a pregnancy test? = Yes

| 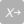 |
| --- |

Q125 Where did you get this test?

- Referral hospital (1)
- Provincial/District hospital (2)
- Health Center (3)
- Health Post (4)
- Outreach (5)
- Community Health Worker (6)
- Other Public Health Facility (7)
- Polyclinic (8)
- Clinic (9)
- Dispensary (10)
- Pharmacy (11)
- Family planning clinic (12)
- Other private medical sector (13)
- Kiosk/Shop/Bar (14)
- Church (15)
- Friend/Relative (16)
- Youth Center (17)
- Other, specify (18) ________________________________________________

Display This Question:

If [DO NOT READ] Sex of the respondent  = Female

And And How old were you at your last birthday?      Text Response Is Greater Than or Equal to 15

And And How old were you at your last birthday?      Text Response Is Less Than or Equal to 19

| 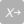 |
| --- |

Q126 Have you ever had abortion?

- Yes, medical (1)
- Yes, surgical (2)
- No (3)
- Refuse to Answer (4)

End of Block: 6. FPRH Service Utilization

Start of Block: 7. Self-efficacy and Intentions

Q127 **Section 7: Self-efficacy and Intentions**

Q128 [READ TEXT ALOUD]

 In this section, we will ask you about how you feel about communicating about contraception and accessing services if you need them. Please ask me if anything is unclear.

| 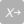 |
| --- |

Q129 **[READ TEXT ALOUD]**

 Now I will read out a few statements about how you communicate with current or if no current partner, your most recent partner. Please tell me your answer using a scale from Strongly Agree to Strongly Disagree
 **[READ STATEMENTS AND OPTIONS ALOUD]**

|  | Strongly agree (1) | Agree (2) | Neither agree nor disagree (3) | Disagree (4) | Strongly disagree (5) |
| --- | --- | --- | --- | --- | --- |
| I can initiate conversations about using contraception with my partner(s). (2) |  |  |  |  |  |
| I know what to do if I am a victim of sexual or gender-based violence (E.g. If someone physically hurt you, hit you, slap you, or sexually assaulted you) (3) |  |  |  |  |  |
| I am confident that I can access and use contraceptives services if I need them (4) |  |  |  |  |  |
| [DO NOT READ] Sex of the respondent  = Female  I know what tools are available to track my menstrual cycle (7) |  |  |  |  |  |

| 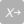 |
| --- |

Q130 **[READ TEXT ALOUD]**

 Now I will read out a few statements about how confident you feel about a few things such as getting your partner to use contraceptives/condoms. These could be hypothetical scenarios but give us your answer thinking about if you had to do it, would you have confidence? 
 Please tell me your answer using the scale from Confident, Somewhat Confident, Unsure, Not Confident.  

 Please note that all your answers are confidential and none of these answers will impact your homework, assignments or grades or performance in school.  [READ QUESTIONS AND OPTIONS ALOUD]

|  | Confident (1) | Somewhat confident (2) | Unsure (3) | Not confident (4) |
| --- | --- | --- | --- | --- |
| How confident are you that you could get your partner to use contraceptives/condoms if you desired it? (1) |  |  |  |  |
| How confident are you that you can get tested for HIV if you need it? (2) |  |  |  |  |
| How confident are you that you can get treatment services for sexually transmitted diseases (other than HIV) if you need it? (3) |  |  |  |  |

Display This Question:

If [DO NOT READ] Sex of the respondent  = Female

And And How old were you at your last birthday?      Text Response Is Greater Than or Equal to 15

And And How old were you at your last birthday?      Text Response Is Less Than or Equal to 19

| 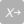 |
| --- |

Q131 We would like to ask you about your current thoughts and feelings about the idea of becoming pregnant in the next 3 months and having a baby in the next year. Women have very different thoughts and feelings about pregnancy and having a baby that can change over time. There are no right or wrong answers. For each question, choose one answer that seems right at this point in your life.    The first set of  questions ask about your thoughts and feelings about the idea of becoming PREGNANT in the next 3 months. Even if you do not think you can become pregnant, please imagine how you would feel about becoming pregnant.   [READ STATEMENTS AND OPTIONS ALOUD]

|  | Strongly Agree (1) | Agree (2) | Neither Agree nor Disagree (3) | Disagree (4) | Strongly Disagree (5) |
| --- | --- | --- | --- | --- | --- |
| I wouldn’t mind it if I became pregnant in the next 3 months (1) |  |  |  |  |  |
| It would be a good thing for me if I became pregnant in the next 3 months (2) |  |  |  |  |  |
| Thinking about becoming pregnant in the next 3 months makes me feel unhappy (3) |  |  |  |  |  |
| Thinking about becoming pregnant in the next 3 months makes me feel excited (4) |  |  |  |  |  |
| Becoming pregnant in the next 3 months would bring me closer to my main partner (By main partner, we mean the romantic partner that is the most serious to you. If you don’t have a romantic partner, please think about the person with whom you last had sexual relations.) (5) |  |  |  |  |  |

Display This Question:

If [DO NOT READ] Sex of the respondent  = Female

And And How old were you at your last birthday?      Text Response Is Greater Than or Equal to 15

And And How old were you at your last birthday?      Text Response Is Less Than or Equal to 19

| 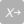 |
| --- |

Q132 The following questions ask about your thoughts and feelings about the idea of having a BABY in the next year. Even if you do not think you can have a baby, please imagine how you would feel about having a baby.
   [READ STATEMENTS AND OPTIONS ALOUD]

|  | Strongly Agree (1) | Agree (2) | Neither Agree nor Disagree (3) | Disagree (4) | Strongly Disagree (5) |
| --- | --- | --- | --- | --- | --- |
| I want to have a baby within the next year (1) |  |  |  |  |  |
| If I had a baby in the next year, it would be bad for my life (2) |  |  |  |  |  |
| It would be a positive addition to my life to have a baby in the next year (3) |  |  |  |  |  |
| It would be the end of the world for me to have a baby in the next year (4) |  |  |  |  |  |
| Thinking about having a baby within the next year makes me smile (5) |  |  |  |  |  |
| Thinking about having a baby within the next year makes me feel stressed out (10) |  |  |  |  |  |
| I would feel a loss of freedom if I had a baby in the next year (11) |  |  |  |  |  |
| If I had a baby in the next year, it would be hard for me to manage raising the child (12) |  |  |  |  |  |
| I would worry that having a baby in the next year would make it harder for me to achieve other things in my life (13) |  |  |  |  |  |

Display This Question:

If [DO NOT READ] Sex of the respondent  = Female

And I want to make certain that I have the correct information. When asked if you had ever had sexual... = Yes

And Are you currently pregnant? = No

Q133 You’re doing a wonderful job so far. Thank you. In this section, we want to know about your intentions to use contraception methods. We will read out a few statements and options. Please give me your honest answer for each statement.

End of Block: 7. Self-efficacy and Intentions

Start of Block: 8. Social and Gender Norms

Q134 Section 8: Social and Gender Norms

Q135 **[READ TEXT ALOUD]** In this section, we will ask you about your views of the society around you. This section explores gender-related norms towards contraception and gender-based violence. Your responses will remain entirely confidential, so please keep this in mind as you answer these questions as honestly as possible. If you have any questions or if anything I ask is unclear, please stop me and I will do my best to give you a better explanation.

| 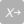 |
| --- |

Q136  Please listen to the following statements and tell me if you strongly agree, agree, neither agree or disagree, disagree, or strongly disagree.

|  | Strongly agree (1) | Agree (2) | Neither agree or disagree (3) | Disagree (4) | Strongly disagree (5) |
| --- | --- | --- | --- | --- | --- |
| Family planning services are only for married men and women or women who have already had children. (9) |  |  |  |  |  |
| Most of my friends think that family planning services are only for married men and women or women who have already had children. (8) |  |  |  |  |  |

End of Block: 8. Social and Gender Norms

Start of Block: 9. Aspirations and Life Goals

Q137 **Section 9: Aspirations and Life Goals**

Q138 **[READ TEXT ALOUD]** 
 In this section, we are interested in hearing about your future aspirations and would like to understand at what age you expect certain things will happen to you in your life. We also want to know if you think the following events will happen to you and if they will happen, when you think they will happen. Please tell me if you think they will happen to you.

|  |
| --- |

Q139 I am confident that I will complete secondary school
 **[READ OPTIONS ALOUD]**

- Strongly Agree (1)
- Agree (2)
- Neither Agree nor Disagree (3)
- Disagree (4)
- Strongly Disagree (5)

|  |
| --- |

Q140 What level do you hope to reach in school?

- S1 (1)
- S2 (2)
- S3 (3)
- S4 (4)
- S5 (5)
- S6 (6)

|  |
| --- |

Q141 Do you hope to get a job outside the home?

- Has already happened (1)
- Will happen (2)
- Will never happen (3)
- Don’t Know (4)

Display This Question:

If Do you hope to get a job outside the home? = Will happen

|  |
| --- |

Q142 If you do get a job, do you hope to be able to save money for your future?

- Yes (1)
- No (2)
- Don't know (3)

|  |
| --- |

Q143 Suppose that on the bottom of this ladder (step 1) stand the poorest people in your community. On the highest step (step 10) stand the wealthiest people in your community. On which step are you today? Please write a number from 1 to 10 below.

________________________________________________________________

|  |
| --- |

Q144 On which step of the ladder do you expect you will be in 10 years, compared to other people in your community?

________________________________________________________________

End of Block: 9. Aspirations and Life Goals

Start of Block: 10. Household Specific Details

Q145 **Section 10: Household Specific Details**

Q146 **[READ TEXT ALOUD]**

 We have almost reached the end of the survey. I would like to ask you last couple of questions related to your home and food security to understand a little bit more about you.

|  |
| --- |

Q147 What type of housing unit does your household usually sleep in?

- House (1)
- Flat / apartment (2)
- Hut (3)
- Tent (4)
- Other, specify (5) ________________________________________________

|  |
| --- |

Q148 Thinking of your home/dwelling, what is the constructional material of the outside walls?

- Mud (1)
- Unburnt/raw bricks (2)
- Stone (3)
- Burnt bricks (4)
- Cement/concrete (5)
- Wood/Bamboo (6)
- Iron sheets (7)
- Cardboard (8)
- Other, specify (9) ________________________________________________
- Don’t Know (10)

|  |
| --- |

Q149 Thinking of your home/dwelling, what is the construction material of the roof of your main dwelling?

- Mud/mud bricks (1)
- Thatch (Grass/Straw) (2)
- Stone (3)
- Burnt bricks (4)
- Corrugated Iron sheets (5)
- Cement/ Concrete (6)
- Roofing tiles (7)
- Asbestos (8)
- Other, specify (9) ________________________________________________
- Don’t Know (10)

|  |
| --- |

Q150 Thinking of your home/dwelling, what is the construction material of the floor of your main dwelling?

- Earth/Mud (1)
- Wood (2)
- Tile (3)
- Concrete (4)
- Straw (5)
- Other, specify (6) ________________________________________________
- Don’t Know (7)

Q151 Now I will talk about your household assets. How many of the following does your household own?   **[NOTE: Read each item below and enter quantity; enter 0 for none or enter -99 for Don't Know]**

- Radio (1) ________________________________________________
- Laptop (4) ________________________________________________
- Feature phone (5) ________________________________________________
- Smart phone (27) ________________________________________________
- Tablet computer (28) ________________________________________________
- Television Set (6) ________________________________________________
- Refrigerator or Freezer (7) ________________________________________________
- Video/ DVD Player (8) ________________________________________________
- Electric/Gas Stove (9) ________________________________________________
- Sofa (10) ________________________________________________
- Motor Vehicle (11) ________________________________________________
- Motorcycle (12) ________________________________________________
- Bicycle (13) ________________________________________________
- Cart (14) ________________________________________________
- Animal-drawn cart (15) ________________________________________________
- Hoes (16) ________________________________________________
- Spraying machine (17) ________________________________________________
- Water pumping set (18) ________________________________________________
- Tractor (19) ________________________________________________
- Trailer for tractors (20) ________________________________________________
- Plough (21) ________________________________________________
- Harrow (22) ________________________________________________
- Milking machine (23) ________________________________________________
- Harvesting and threshing machine (24) ________________________________________________
- Hand-milling machine (25) ________________________________________________
- Wheel Barrow (26) ________________________________________________

Q152
**[READ TEXT ALOUD]**
   The next few questions are intended to understand if you/your family are under strain of resources. Your responses will remain entirely confidential, so please keep this in mind as you answer these questions as honestly as possible. If you have any questions or if anything I ask is unclear, please stop me and I will do my best to give you a better explanation.

|  |
| --- |

Q153 In the past 30 days, was there ever no food to eat of any kind in your house because of lack of resources to get food?

- No (1)
- Yes (2)

Skip To: Q155 If In the past 30 days, was there ever no food to eat of any kind in your house because of lack of r... = No

Display This Question:

If In the past 30 days, was there ever no food to eat of any kind in your house because of lack of r... = Yes

|  |
| --- |

Q154 How often did this happen in the past 30 days?

- Rarely (1-2 times) (1)
- Sometimes (3-10 times) (2)
- Often (More than 10 times) (3)

|  |
| --- |

Q155 In the past 30 days, did you or any household member go to sleep at night hungry because there was not enough food?

- No (1)
- Yes (2)

Skip To: Q157 If In the past 30 days, did you or any household member go to sleep at night hungry because there wa... = No

Display This Question:

If In the past 30 days, did you or any household member go to sleep at night hungry because there wa... = Yes

|  |
| --- |

Q156 How often did this happen in the past 30 days?

- Rarely (1-2 times) (1)
- Sometimes (3-10 times) (2)
- Often (More than 10 times) (3)

|  |
| --- |

Q157 In the past 30 days, did you or any household member go a whole day and night without eating anything at all because there was not enough food?

- No (1)
- Yes (2)

Skip To: End of Block If In the past 30 days, did you or any household member go a whole day and night without eating anyt... = No

Display This Question:

If In the past 30 days, did you or any household member go a whole day and night without eating anyt... = Yes

|  |
| --- |

Q158 How often did this happen in the past 30 days?

- Rarely (1-2 times) (1)
- Sometimes (3-10 times) (2)
- Often (More than 10 times) (3)

End of Block: 10. Household Specific Details

Start of Block: 11. CyberRwanda

Q159 **Section 11: CyberRwanda**

Q160 **[READ TEXT ALOUD]**

 You’ve been such a great respondent. In this last and final section, I would like to ask you some questions about CyberRwanda program.

|  |
| --- |

Q161 Have you heard of CyberRwanda program in your school?

- Yes (1)
- No (2)

Skip To: End of Block If Have you heard of CyberRwanda program in your school? = No

Display This Question:

If Have you heard of CyberRwanda program in your school? = Yes

|  |
| --- |

Q162 What kind of a program is it?

- Education program with Cyber Clubs in schools and an online ordering system for contraceptionEducation program with Cyber Clubs in schools and an online ordering system for contraception (1)
- It’s a new game on the mobile phone (2)
- New Government program (3)
- Other, specify (4) ________________________________________________
- Don’t Know (5)

|  |
| --- |

Q163 Who informed you about this program at school?

- Principal/Head Teacher (1)
- Teacher/School/Youth Center Staff (2)
- Launch event (3)
- Students/Friends (4)
- Pharmacist (5)
- No one (6)
- Other, specify (7) ________________________________________________

|  |
| --- |

Q164 As part of CyberRwanda program, did you use the Cyber Clubs at School?

- Yes (1)
- No (2)
- Don't Know (3)

|  |
| --- |

Q165 As part of CyberProgram, did you use the website outside of school?

- Yes (1)
- No (2)
- Don't know (3)

Display This Question:

If As part of CyberRwanda program, did you use the Cyber Clubs at School? = Yes

Or As part of CyberProgram, did you use the website outside of school? = Yes

|  |
| --- |

Q166 How many times have you used CyberRwanda since it has been available in your school?

________________________________________________________________

Display This Question:

If As part of CyberRwanda program, did you use the Cyber Clubs at School? = Yes

Or As part of CyberProgram, did you use the website outside of school? = Yes

|  |
| --- |

Q167 What did you use it for?

- To learn about contraception (1)
- To order contraceptives (2)
- To plan for my future (3)
- Other, specify (4) ________________________________________________
- Don’t know (5)

Display This Question:

If As part of CyberProgram, did you use the website outside of school? = Yes

|  |
| --- |

Q168 Where did you use it?

- Personal Computer/Laptop (1)
- Personal Smartphone (2)
- Someone else's computer/laptop (3)
- Someone else's smartphone (4)
- Other, specify (5) ________________________________________________

Display This Question:

If As part of CyberRwanda program, did you use the Cyber Clubs at School? = Yes

Or As part of CyberProgram, did you use the website outside of school? = Yes

|  |
| --- |

Q169 Can you name any TWO characters that appear on CyberRwanda platform?   **[DO NOT READ OPTIONS ALOUD, SELECT ALL THAT APPLY]**

- Mutoni (1)
- Ntwali (2)
- Happy (3)
- Munezero (4)
- Nurse Keza (5)
- Mama (6)
- Other, specify (7) ________________________________________________

Display This Question:

If As part of CyberRwanda program, did you use the Cyber Clubs at School? = Yes

Or As part of CyberProgram, did you use the website outside of school? = Yes

|  |
| --- |

Q170 Which parts of the program do you use most frequently?
 **[SELECT ONE OPTION]**

- Ask Mutoni/Ntwali (1)
- Online ordering (2)
- Stories/vignettes (3)

Display This Question:

If As part of CyberRwanda program, did you use the Cyber Clubs at School? = Yes

Or As part of CyberProgram, did you use the website outside of school? = Yes

|  |
| --- |

Q171 What did you learn from the character, Mutoni?   **[SELECT ALL THAT APPLY]**

- Writing a resume (1)
- How to do networking (2)
- Managing money wisely (3)
- About abusive relationships (4)
- Other, specify (5) ________________________________________________

Display This Question:

If As part of CyberRwanda program, did you use the Cyber Clubs at School? = Yes

Or As part of CyberProgram, did you use the website outside of school? = Yes

|  |
| --- |

Q172 What did you learn from the character, Mama?

- Puberty (1)
- Menstruation/periods (2)
- What to do if you are having trouble in school (3)
- Other, specify (4) ________________________________________________

Display This Question:

If As part of CyberRwanda program, did you use the Cyber Clubs at School? = Yes

Or As part of CyberProgram, did you use the website outside of school? = Yes

|  |
| --- |

Q173 Have you specifically used the online ordering feature?

- Yes (1)
- No (2)

Skip To: Q179 If Have you specifically used the online ordering feature? = No

Display This Question:

If Have you specifically used the online ordering feature? = Yes

|  |
| --- |

Q174 What did you order?
 
**[SELECT ALL THAT APPLY]**

- Birth Control Pill (1)
- Condoms (male) (2)
- Condoms (female) (3)
- Emergency contraceptives (4)
- Pads (5)
- Painkillers (6)
- Pregnancy test (7)
- Other, specify (8) ________________________________________________

Display This Question:

If Have you specifically used the online ordering feature? = Yes

|  |
| --- |

Q175 How easy was it to use 'the online ordering feature'?  
 
**[READ OPTIONS ALOUD]**

- Very Easy (1)
- Easy (2)
- Neither Easy Nor Difficult (3)
- Difficult (4)
- Very Difficult (5)

Display This Question:

If How easy was it to use 'the online ordering feature'?     [READ OPTIONS ALOUD] = Difficult

And How easy was it to use 'the online ordering feature'?     [READ OPTIONS ALOUD] = Very Difficult

|  |
| --- |

Q176 What were some challenges you faced when ordering online?

- It did not work (1)
- Payment option did not go through (2)
- Other, specify (3) ________________________________________________

|  |
| --- |

Q177 Did you have any challenges while picking up your order at the pharmacy?

- Yes (1)
- No (2)

Display This Question:

If Did you have any challenges while picking up your order at the pharmacy? = Yes

|  |
| --- |

Q178 What specific challenges did you face while ordering at the pharmacy?

- Pharmacy was closed (1)
- Pharmacist was rude/unhelpful (2)
- Pharmacist did not keep the order ready (3)
- Other (Specify) (4) ________________________________________________

|  |
| --- |

Q179 Have you talked with anyone about the CyberRwanda Program?

- Yes (1)
- No (2)

Display This Question:

If Have you talked with anyone about the CyberRwanda Program? = Yes

|  |
| --- |

Q180 Who have you talked with about the CyberRwanda Program?

- Parents (1)
- Friends who go to your school (2)
- Friends who go to other schools (3)
- Siblings (4)
- Teachers (5)
- Family members (6)
- None (7)
- Other, specify (8) ________________________________________________

|  |
| --- |

Q181 Should your school continue to receive this CyberRwanda Program?

- Yes (1)
- No (2)

|  |
| --- |

Q182 Do you think all schools in your district should have the CyberRwanda Program?

- Yes (1)
- No (2)

Display This Question:

If Have you heard of CyberRwanda program in your school? = Yes

And Have you specifically used the online ordering feature? = Yes

Or As part of CyberRwanda program, did you use the Cyber Clubs at School? = Yes

|  |
| --- |

Q183 Would you be wiling to be interviewed by one of our study staff to talk more about your experience using CyberRwanda?

- Yes (1)
- No (2)

End of Block: 11. CyberRwanda

Start of Block: 4B. Sexual Behavior (MALES)

Q184 **Section 4B: Sexual Behavior (MALES)**

|  |
| --- |

Q187 Has any girl/female ever become pregnant by you, or told you that she became pregnant by you, regardless of the outcome of the pregnancy or if no child was born?

- Yes (1)
- No (2)
- Don’t know (3)
- Refuse to answer (4)

Q188
How old were you the first time you had vaginal sex/sexual intercourse?
 

- 12 years old or younger (5)
- 13 years (6)
- 14 years (7)
- 15 years (8)
- 16 years (9)
- 17 years (10)
- 18 years (11)
- 19 years (12)
- Never had sex (13)

Skip To: End of Block If How old were you the first time you had vaginal sex/sexual intercourse?   = Never had sex

|  |
| --- |

Q189 The first time you had sexual intercourse with someone would you say you were willing, somewhat willing, or not willing at all to? Willing means you gave permission or said it was OK, or that you did it because you wanted to and not because someone made you.
[READ OPTIONS ALOUD]

- Very willing (1)
- Somewhat willing (2)
- Not willing at all (3)
- Refuse to answer (4)

|  |
| --- |

Q190 Have you ever had sex in exchange for money, food, gifts, drugs, alcohol, shelter or other goods?

- Yes (1)
- No (2)
- Don’t know (3)
- Refuse to answer (4)

Q191 [READ TEXT ALOUD]
Now I’m going to ask you to think about your current partner, or if you don’t currently have a partner, your most recent partner.

|  |
| --- |

Q192 When was the last time you had sexual intercourse with this partner?


[READ OPTIONS ALOUD]

- Within the last 6 months (1)
- 6-12 months ago (2)
- A year or more ago (3)

|  |
| --- |

Q193 Did you ever discuss using contraception with this partner?

- Yes (1)
- No (2)
- Refuse to answer (3)

|  |
| --- |

Q194 The last time you had sex with this partner, did you do anything to avoid pregnancy?

- Yes (1)
- No (2)
- Don’t Know (3)
- Refuse (4)

Display This Question:

If The last time you had sex with this partner, did you do anything to avoid pregnancy? = Yes

|  |
| --- |

Q195 What did you use?
 
[READ OPTIONS ALOUD. SELECT ALL THAT APPLY]

- Pills (1)
- Condoms (2)
- Female Condoms (3)
- IUD / Intrauterine devices (4)
- Injectables (5)
- Implants/ Jadelle (6)
- Emergency Contraceptive Pills (7)
- Diaphragm (8)
- Foam/Jelly (9)
- Standard Days Method (10)
- Female Sterilization (11)
- Male Sterilization (12)
- Lactational Amenorrhea Method (LAM) (13)
- Periodic Abstinence (14)
- Withdrawal (15)
- Nothing (16)
- Misoprostol (17)
- Other (Specify) (18) ________________________________________________
- Don’t know (19)
- Refuse to answer (20)

Display This Question:

If The last time you had sex with this partner, did you do anything to avoid pregnancy? = Yes

|  |
| --- |

Q196 Where did you or your partner get this method?     [READ OPTIONS ALOUD]   **[NOTE: Probe to identify the type of source. If unable to determine if public or prvate sector, write the name of the place]**

- Referral hospital (1)
- Provincial/District hospital (2)
- Health Center (3)
- Health Post (4)
- Outreach (5)
- Community Health Worker (6)
- Other Public Health Facility (7) ________________________________________________
- Polyclinic (8)
- Clinic (9)
- Dispensary (10)
- Pharmacy (11)
- Family planning clinic (12)
- Other private medical sector (13)
- Kiosk/Shop/Bar (14)
- Church (15)
- Friend/Relative (16)
- Youth Center (17)
- Other, specify (18) ________________________________________________

Display This Question:

If The last time you had sex with this partner, did you do anything to avoid pregnancy? = No

Or The last time you had sex with this partner, did you do anything to avoid pregnancy? = Don’t Know

Or The last time you had sex with this partner, did you do anything to avoid pregnancy? = Refuse

|  |
| --- |

Q197 Young people have different reasons for not using contraception. Tell me which reasons best describe why you and your partner are not currently using contraception, or why you didn’t use contraception with your most recent partner. 

 [DO NOT READ OPTIONS. SELECT ALL THAT APPLY]   [NOTE: **If no current partner, insert “why didn’t you use contraception with your last partner”]**

- I am too embarrassed to talk about using contraception (1)
- I want to get pregnant (2)
- It is too hard to get my partner to use contraception with me (3)
- Contraception interferes with enjoyment (4)
- I don’t know where to get contraception (5)
- I don’t want to seem too eager for sex (6)
- I don’t think I could get pregnant (7)
- I have never really thought about it (8)
- I can’t afford it (9)
- I’m embarrassed to buy it (10)
- People will judge me for using it/buying it (11)
- Other (12)
- Refuse to answer (13)

|  |
| --- |

Q198
Now still thinking about the last time you had sex with this partner, were you aware if your partner used anything to delay or avoid pregnancy?

- Yes (1)
- No (2)
- Don't know (3)
- Refuse (4)

| Page Break |  |
| --- | --- |

End of Block: 4B. Sexual Behavior (MALES)

Start of Block: End of Survey

Q199 **END OF SURVEY**

Q200 Thank you very much for your time today, and please remember that everything you told me is private and confidential and won’t be shared with anyone outside the study investigators. Please remember this survey has nothing to do with your classes or homework or grades. Nothing you told me here will affect any of your school performance. 
Is there anything else you want to tell me?

End of Block: End of Survey

Start of Block: NOTES

Q201 **NOTES

 Instructions:**Write any notes that may be needed to interpret or analyze the data collected. Specify the section and question referenced.

________________________________________________________________

________________________________________________________________

________________________________________________________________

________________________________________________________________

________________________________________________________________

End of Block: NOTES

Start of Block: Unplaced questions

Start of Block: Block 19
