# Supplementary material for: Design and impact evaluation of a digital reproductive health program in Rwanda using a cluster randomized design: study protocol
Source: BMC Public Health. 2020 Nov 13;20:1701. doi: 10.1186/s12889-020-09746-7 (PMC7662730; doi:10.1186/s12889-020-09746-7)
Supplement: Supplementary file 4 — Additional file 4. Model Consent Forms. Model consent form and other related documentation given to participants and authorized surrogates. [file 12889_2020_9746_MOESM4_ESM.docx]

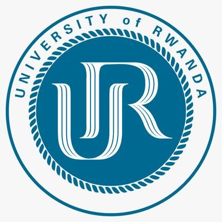

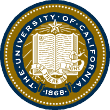


CONSENT FOR CHILDREN (18 YEARS AND ABOVE)

TO PARTICIPATE IN RESEARCH

*Impact Evaluation of CyberRwanda in Rwanda*

Eligibility Checklist

___ Age 18 or 19

___ Enrolled in S3 or S4

**Key Information**

- You are being invited to participate in a research study. Participation in research is completely voluntary.
- The purpose of the study is to understand the health and well-being among students in schools in this district of Rwanda.
- This is an 18-month study. You will be asked to complete a questionnaire administered by one of our research staff at the beginning of the study (in about one week), in the middle of the study (about 9 months from now) and at the end of the study (about 18 months from now). We may contact you again to participate in a semi-structured interview as well.
- There is no direct benefit to you but the we hope that the results of the research will help improve ways of using digital tools to reach youth/students with information on their health and well-being in the future.

**Introduction**

My name is ___________________ and I am working for __________ at Society for Family Health in Kigali and ______ at YLabs based in Kigali and Berkeley, California. SFH and YLabs are organizations working together to improve the health of young people in Rwanda. I am also working with Professor Sandra McCoy at the University of California, Berkeley and Dr. Aline Umubyeyi at the University of Rwanda to conduct a research study. We invite you to participate in this study.

Before you decide whether to be part of this study, it’s important for you to understand why we’re doing the research and what’s involved. Please read this form carefully. If you have any questions about the research, feel free to ask us.

**Purpose**

We are doing this study to understand more about the health and well-being of students between the ages of 12 and 19 who are enrolled in secondary schools in levels S3 and S4 across several districts in Rwanda. We are inviting you to participate because you are in this age range, attend one of the study schools, and are currently enrolled in level S3 or S4 at one of the study schools. The school has agreed that we can conduct the research here. This study is not part of your school work and it will not be graded.

# Procedures

If you agree to be in this study, and your parents give permission, we will ask you to:

- Respond to a baseline questionnaire

We will ask you to respond to a questionnaire that will be administered by a research assistant who is part of our study staff. The research assistant will ask you a set of questions and then fill out your responses on a computer tablet. The questionnaire will take about 30-45 minutes. The survey is generally about your health and well-being and includes some questions on sensitive topics including sexual behavior and health history, and work-related questions about employment and wages.

- Provide contact information

We will also ask you to provide contact information so that we can reach you again for the follow-up surveys if you are no longer enrolled at the study schools.

- Respond to a midline questionnaire (9 months from now)

We will contact you again in approximately 9 months and ask you to complete another questionnaire covering similar topics to the first survey. This second questionnaire will follow the same procedures at the first one, and will also take about 30-35 minutes to complete.

- Respond to an endline questionnaire (18 months from now)

We will contact you again in approximately 18 months and ask you to complete another questionnaire covering similar topics to the first and second surveys. This third and final questionnaire will follow the same procedures at the first one, and will also take about 30-35 minutes to complete.

- Request permission to contact you for other studies

We will collect your contact information, including name, phone number, and address, in case we need to contact you to clarify information and to see if you are interested in talking with us again about this study or about other studies in the future.

**Study Location and Time:** We will do the surveys at a private place at your school, or if you are no longer in school for the future surveys, we will find a private place of your choosing to conduct the survey. Each survey will take about 30-45 minutes of your time.

**Benefits**

There is no benefit to you personally for taking part in this study. However, we hope that the results of the research will help improve ways of reaching youth/students with information on their health and well-being in the future.

# Risks/Discomforts

- You may get bored or tired and decide that he or she does not want to complete the study activities. If so, your child can just tell us that he or she wishes to stop.
- You may feel uncomfortable with some of the questions we ask you. If so, you can ask to skip a question, or you can feel free to stop at any time.
- *Breach of Confidentiality*: A possible risk for any research is that confidentiality could be compromised, that is, people outside the study might get hold of confidential study information. We will do everything we can to minimize this risk.

**Confidentiality**

We will keep your study data as confidential as possible. If we publish or present results of this study, we will not use individual names or other personally identifiable information. All the data will be handled as confidentially as possible. To protect confidentiality, we will do the following:

- Any study information in the study database will not include your name and contact information. All of your information will instead be identified with a random code.
- Your research records will always be securely stored. Paper forms that contain personal identifying information will be stored in a locked cabinet in a locked office in a secured building. Computer databases that contain your data will be stored in an encrypted format on password-protected computers and will require a password so that only authorized study personnel have access.
- Publications or presentations with results of this study will not use individual names or any other personally identifiable information.

## Future use of study data

The research data will be maintained for possible use in future research by the research team or other collaborators. We will retain this data for up to 10 years after the study is over. The same measures described above will be taken to protect confidentiality of this study data.

**Compensation/Payment**

You will not be paid for being in this study.

Rights

***Participation in research is completely voluntary****.* Research is something you do only if you want to. No one will get mad at you if you don’t want to be in the study. And whether you decide to participate or not, either way will have no effect on your grades at school. And remember, you can always change your mind later if you don't want to be in the study any more.

**Do you have any questions?**

You can contact us if you have questions about the study, or if you decide you don’t want to be in the study any more. You can talk to me, or your parents, or someone else at any time during the study if you like.

You can contact [Name] from SFH at [phone number] or email**:** [[email]](mailto:jturatsinze@sfhrwanda.org)

Or you can contact [Name] in the United States at [phone number] or [[email]](mailto:smccoy@berkeley.edu).

If you have any questions or concerns about your child’s rights and treatment as a research subject, you may contact the Rwanda National Ethics Board at [phone number] or the office of the University of California, Berkeley's Committee for the Protection of Human Subjects, at [phone number] or [[email].](mailto:subjects@berkeley.edu)

******************************************

**CONSENT OF ADOLESCENT (18 years old and above)**

If you decide to participate in this study, ***please sign and date below***. We will give you a copy of this form to keep for future reference.

___________________________________

Participant Name (*please print*)

_______________

Participant Signature Date

_______________

Signature of Investigator/Person Obtaining Consent Date


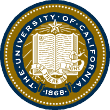

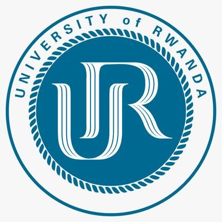


**ASSENT TO PARTICIPATE IN A RESEARCH STUDY**

*Impact Evaluation of CyberRwanda in Rwanda*

*Children/Youth <18 Years Old*

Eligibility Checklist

___ Age 12 - 17

___ Enrolled in S3 or S4

___Parent/guardian did not opt out

**Introduction**

My name is ___________________ and I am working for _______at YLabs and _________ at Society for Family Health in Kigali and ______ at YLabs based in Kigali and Berkeley, California. SFH and YLabs are organizations working together to improve the health of young people in Rwanda. I am also working with Professor Sandra McCoy at the University of California, Berkeley and Dr. Aline Umubyeyi at the University of Rwanda to conduct a research study. We invite you to participate in this study.

**What is a research study?**

A research study is when people collect information to find out more about an important issue or topic. Before you decide if you want to be in this study, it’s important for you to understand why we’re doing the research and what’s involved.

Please read this form carefully. You can discuss it with your parents, teachers, or anyone else. If you have questions about this research, you can also ask me.

**Why are we doing this study?**

We are doing this study to understand the health and wellbeing of students in schools in this district of Rwanda. This study is not part of your school work, and you won't be graded on it.

**Why are we talking to you about this study?**

We’re inviting you to take part because you go to a school where we are doing the study, are in level S3 or S4, and belong to the age group we are interested in studying.

**What will happen if you are in this study?**

If you agree to be in this study, and your parents give permission, we will ask you to:

- Complete up to three questionnaires; today, 9 months from now, and 18 months from now

We will ask you to complete up to three questionnaires that will be administered by a research assistant who is part of our study staff. The first questionnaire will happen today. The second will be about 9 months from now and the third will be about 18 months from now. In each questionnaire, the research assistant will ask you a set of questions and then fill out your responses on a computer tablet. The questionnaires will take about 30-45 minutes. Each questionnaire is generally about your health and well-being and includes some questions on sensitive topics including sexual behavior and health history, and work-related questions about employment and wages.

- Provide contact information for follow-up surveys

We will also ask you to provide several ways to contact you so that we can reach you again for the follow-up questionnaires if you are no longer enrolled at the study schools.

- Request permission to contact you for other studies

We will collect your contact information, including name, phone number, and address, in case we need to contact you to clarify information and to see if you are interested in talking with us again about this study or about other studies in the future.

**Study Location and Time:** We will do the surveys at a private place at your school, or if you are no longer in school for the future surveys, we will find a private place of your choosing to conduct the survey. Each survey will take about 30-45 minutes of your time.

**Are there any benefits to being in the study?**

There are no benefits to you personally for taking part in this study, but we hope that the results of the research will help to improve the health and well-being of youth in the future. This research will also help us design new digital health tools for adolescents.

**Are there any risks or discomforts to being in the study?**

- You might decide that you don’t want to finish the questionnaire. If so, just tell us that you want to stop and we will end the interview.
- You may feel uncomfortable with some of the questions. If so, you can ask to skip a question, or you can feel free to stop at any time. Keep in mind that anything you tell us is completely confidential.
- A possible risk for any research is that people outside the study might obtain confidential study information. We will do everything we can to make sure that doesn't happen.

**Who will know about your study participation?**

The researchers are the only ones who will know the details of your study participation. If we publish reports or give presentations about this research, we will only discuss group results. We will not use your name or any other personal information that would identify you.

To help protect confidentiality, we will give your study data a code number, and protect the data in a file with a password that only the researchers know. Any paper forms that contain your name and other personal identifying information will be stored in a locked cabinet in a locked office in a secured building.

After the study is complete, we plan to keep this information for 10 years, in case we or other researchers want to use study information for other studies. In this case, we will follow the same steps we just described to keep it as confidential as possible.

**Will you get paid for being in the study?**

You will not be paid for being in this study.

**Do you have to be in the study?**

No, research is voluntary. No one will get mad if you don’t want to be in the study. And whether you decide to participate or not, either way will have no effect on your grades at school. And remember, you can always change your mind later if you don't want to be in the study any more.

**Do you have any questions?**

You can contact us if you have questions about the study, or if you decide you don’t want to be in the study. You can talk to me, your parents, or someone else at any time during the study if you like.

You can contact [Name] from SFH at [phone number] or email**:** [[email]](mailto:jturatsinze@sfhrwanda.org)

Or you can contact [Name] in the United States at [phone number] or [[email]](mailto:smccoy@berkeley.edu).

If you have any questions or concerns about your child’s rights and treatment as a research subject, you may contact the Rwanda National Ethics Board at [phone number] or the office of the University of California, Berkeley's Committee for the Protection of Human Subjects, at [phone number] or [[email].](mailto:subjects@berkeley.edu)

******************************************

**ASSENT OF ADOLESCENT (12–17 years old)**

If you decide to participate, and your parents do not decline your participation we'll give you a copy of this form to keep for future reference.

**If you would like to be in this research study, please sign your name on the line below.**

________________________________________ _______________

Child's Name/Signature (*printed or written by child*)* Date

________________________________________ _______________

Signature of Investigator/Person Obtaining Assent Date

******************************************

**If verbal assent only is being obtained:*

Investigator or Person Conducting Assent Discussion: Initial here if child cannot sign, to document that child received this information and gave assent verbally: ______


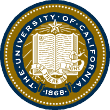

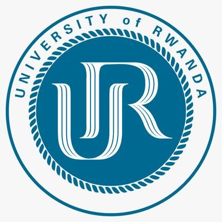
PARENT INFORMATION FORM: RESEARCH AT YOUR CHILD’S SCHOOL

*Evaluation of CyberRwanda intervention in Rwanda*

**Key Information**

- Your child is being invited to participate in a research study. Participation in research is completely voluntary.
- The purpose of the study is to understand the health and well-being among students in schools in this district of Rwanda.
- This is an 18-month study. Your child will be asked to complete a questionnaire administered by one of our research staff at the beginning of the study (in about one week), in the middle of the study (about 9 months from now) and at the end of the study (about 18 months from now).
- Each questionnaire will take about 30-45 minutes for your child to complete.
- There are no direct benefits to your child but the we hope that the results of the research will help improve how we reach youth with information on their health and well-being in the future.
- **If you wish to allow your child to participate in the research study no action is necessary**

**Introduction**

We are requesting the participation of your child in a research study that is taking place at their school. This research is being conducted by Dr. Aline Umubyeyi at the University of Rwanda and Dr. Sandra McCoy at the University of California Berkeley, in partnership with Society for Family Health and YLabs in Kigali. Before you and your child decide whether he/she will be part of this study, it’s important for all of you to understand why we’re doing the research and what is involved. Please read this information carefully. (Your child will receive his/her own assent form.) We encourage you to discuss the study with your child. If you or your child has questions about the research, feel free to ask us.

**Purpose**

We are doing this study to understand more about the health and well-being of students between the ages of 12 and 19 who are enrolled in secondary schools in levels S3 and S4 across several districts in Rwanda. We are inviting your child to participate because he/she is in this age range, attends one of the study schools, and is currently enrolled in level S3 or S4 at one of the study schools. The school has agreed that we can conduct the research there. This study is not part of your child’s school work and it will not be graded.

# Procedures

If your child decides to participate and you do not decline his/her participation, we will ask him/her to:

- Complete up to three questionnaires; today, 9 months from now, and 18 months from now

We will ask your child to complete up to three questionnaires that will be administered by a research assistant who is part of our study staff. The first questionnaire will in about a week. The second will be about 9 months from now and the third will be about 18 months from now. In each questionnaire, the research assistant will ask your child a set of questions and then fill out his/her responses on a computer tablet. The questionnaires will take about 30-45 minutes. Each questionnaire is generally about your child’s health and well-being and will include some questions on sensitive topics including sexual behavior and health history, and work-related questions about employment and wages.

- Provide contact information:

We will ask your child to provide contact information so that we can reach them again for the follow-up questionnaires, should they no longer be enrolled at the study schools.

- Request permission to contact you for other studies

We will collect your child’s contact information, including name, phone number, and address, in case we need to contact him/her to clarify information and to see if he/she is interested in talking with us again about this study or about other studies in the future.

**Study Location**

All the study procedures will take place at your child’s school. If your child has discontinued school for the future questionnaires, we may ask to survey your child at your home or at another private location of your child’s choosing.

**Benefits**

There is no benefit to you or your child personally for taking part in this study. However, we hope that the results of the research will help improve how we reach youth with information on their health and well-being in the future.

**Risks/Discomforts**

- Your child might decide that s/he does not want to complete the study questionnaire. If so, your child can tell us and we will end the interview.
- Your child may feel uncomfortable with some of the questions. If so, your child can ask to skip a question, or can feel free to stop the interview at any time.
- *Breach of Confidentiality*: A possible risk for any research is that confidentiality could be compromised, that is, people outside the study might obtain confidential study information. We will do everything we can to minimize this risk.

**Confidentiality**

We will keep your child's study data as confidential as possible. If we publish or present results of this study, we will not use individual names or other personally identifiable information. All the data will be handled as confidentially as possible. To protect confidentiality, we will do the following:

- Any study information in the study database will not include your child’s name and contact information. All of your child’s information will instead be identified with a random code.
- Your child’s research records will always be securely stored. Paper forms that contain personal identifying information will be stored in a locked cabinet in a locked office in a secured building. Computer databases that contain your child’s data will be stored in an encrypted format on password-protected computers and will require a password so that only authorized study personnel have access.
- Publications or presentations with results of this study will not use individual names or any other personally identifiable information.
- We might use your child’s information for future research studies or distribute the data to other investigators for future research studies without additional informed consent from the subject, the parent, or the legally authorized representative. If we do so, all of your child’s identifying information will be removed from the data.

**Compensation/Payment**

You/your child will not be paid for being in this study.

Rights

***Participation in research is completely voluntary****.* You have the right to decline your child’s participation or to withdraw your child at any point from this study without penalty or loss of benefits to which you are otherwise entitled. Your child has the same rights to decline to participate or withdraw from the study at any time.

**Questions**

You can contact [Name] from SFH at [phone number] or email**:** [[email]](mailto:jturatsinze@sfhrwanda.org)

Or you can contact [Name] in the United States at [phone number] or [[email]](mailto:smccoy@berkeley.edu).

If you have any questions or concerns about your child’s rights and treatment as a research subject, you may contact the Rwanda National Ethics Board at [phone number] or the office of the University of California, Berkeley's Committee for the Protection of Human Subjects, at [phone number] or [[email].](mailto:subjects@berkeley.edu)

******************************************

**If you wish to allow your child to participate in the research study no action is necessary.**

**If you do not allow your child to take part in the study, do one of the following by (date______):**

(1) Call the study staff – [Name] - at [phone number]

(2) SMS the study staff at [phone number] and include your name and your child’s name in the message. We will respond with a confirmation that your request was received.
