# Supplementary material for: Design and impact evaluation of a digital reproductive health program in Rwanda using a cluster randomized design: study protocol
Source: BMC Public Health. 2020 Nov 13;20:1701. doi: 10.1186/s12889-020-09746-7 (PMC7662730; doi:10.1186/s12889-020-09746-7)
Supplement: Supplementary file 5 — Additional file 5. Data Management and Monitoring Protocols. Detailed methods on data management, monitoring, and dissemination, in compliance with SPIRIT guidelines. [file 12889_2020_9746_MOESM5_ESM.docx]

**Data Management and Monitoring Protocols**

**Data Management:**Plans for data entry, coding, security, and storage.

- Participant surveys will be administered using Qualtrics Offline Surveys on password-protected tablet computers. Data from the questionnaires will be uploaded to a password-protected cloud-based storage folder and removed from the individual tablets on a daily basis. Only key members of the study staff will have access to the cloud-based storage folder. Study participant logs linking unique study ID to the participant’s name and contact information will be stored separately from the survey data, in a password-protected file accessible only by relevant study staff when contacting the participants is necessary for follow-up. Once follow-up is complete, the log linking unique study ID to participant name and contact information will be destroyed. In terms of data storage, in the spirit of open access, we will explore anonymizing the dataset and allowing public access in collaboration with the Ministry and other in-country partners.

**Harms:**Plans for collecting, assessing, reporting, and managing solicited and spontaneously reported adverse events and other unintended effects of trial interventions or trial conduct.

- Potential anticipated adverse events include breach of confidentiality resulting in accidental disclosure of a participants’ survey responses, or other unforeseen consequences.
- We will follow the reporting requirements outlined in section 1.3 of the Committee for the Protection of Human Subjects at the University of California, Berkeley (CPHS) Unanticipated Problems and Adverse Events Policies and Procedures document (RR408). Specifically, unanticipated serious adverse events will be reported to the IRB by fax, mail/delivery, phone, or email within 1 week (7 calendar days) of the investigators becoming aware of the event. The initial report will be followed by a formal written report within no more than two weeks (14 calendar days) of the Principal Investigator learning of the incident. We will also report any severe adverse events that are definitely or probably related to the interventions to the Rwanda National Ethics Committee (RNEC), a regulatory body that has/will approve this protocol in addition to CPHS at the University of California, Berkeley.
- Additionally, we will report to the funders the following events: adverse events, serious adverse events, unanticipated problems involving risks to subjects or others, protocol violations, non- compliance, or suspension or terminations by monitoring entities. These reports will indicate that the monitoring entities (i.e., the PI, CPHS, and RNEC) have been notified in accordance with the approved monitoring plan and federal regulations.
- Adverse events will be identified by participant report, follow-up interviews, or report by school staff. Participants will be asked about any adverse effects during their follow-up study visits at 9 and 18 months.
- To attribute adverse events to study procedures and/or the interventions being tested, we will use the following set of categories: Directly related to the research, Indirectly related to the research, Unrelated to the research.

**Auditing:**Frequency and procedures for auditing trial conduct.

- Survey data will be reviewed for quality and completeness by team supervisors before the end of each day of data collection. Each team will have a supervisor who is responsible for accompanying each surveyor in his/her team for the entire day. This team supervisor will only be responsible for conducting a small number of surveys – their primary responsibility will be to ensure data is being collected in accordance with the protocol and to troubleshoot challenges in real-time. At the end of each day, the team supervisor will collect all the tablets and notebooks of the surveyors under his/her supervision and verify the data collected before uploading the data from the tablets to the cloud-based database. The study team will produce a weekly report listing missing data and skipped questions and identify if these errors are associated with particular field team members. Those field team members will then be flagged for supportive supervision, additional training, or release from the team. The tablets will then be returned to the team supervisors, who will distribute them among the surveyors under their supervision to continue data collection. Surveyors will be redeployed to correct and/or complete surveys that are incomplete. Quality indicators will be discussed on every team call once data collection has commenced.

**Protocol Amendments:**Plans for communicating important protocol modifications to relevant parties.

- Protocol amendments will be submitted through both the University of California Berkeley IRB and the Rwanda IRB. Any important changes approved by the IRBs and as listed above will be communicated to all investigators and study stakeholders, and if relevant, to study participants. Changes will also be updated to [clinicaltrials.gov](http://clinicaltrials.gov/) and through our pre-specified analysis plan at AsPredicted.

**Confidentiality:**How personal information about potential and enrolled participants will be collected, shared, and maintained in order to protect confidentiality before, during, and after the trial.

- We will adhere to strict measures to ensure the confidentiality of the data after the interviews are complete (as outlined in our ethical review board applications to the Rwanda National Ethics Committee (RNEC, Rwanda) and the Center for the Protection of Human Subjects (University of California, Berkeley)). In addition, YLabs has a local office study personnel in Kigali who adhere to well-established data collection and management systems. A key policy of the research group is that the fewer individuals handling sensitive information, the greater the protection. Thus, we will adhere to the following guidelines:
- Project files and databases will only be available to research personnel through the authorization of the Principal Investigator (Dr. McCoy). Access to any of the data will be limited to the Principal Investigator, relevant mentors and advisors, and other key personnel, and require a password at all times.
- All staff with access to the data will sign confidentiality agreements to maintain confidentiality even after they have left the study or the study has ended.
- The team will have a standard operating procedure in place to conduct quality control spot checks and ensure the privacy and confidentiality of study participants, data collection forms, and electronic data.
- Staff will be trained on the policies and procedures for data management and transmission and will receive instructions on how to report any violations of those policies and procedures.
- All staff will be appropriately trained in the handling of sensitive study data through their employment training and annual completion of training in the ethical conduct of research (University of California Berkeley staff and students).
- The Principal Investigator will review all procedures for protecting confidentiality with study staff on a bi-monthly basis, including storing the data and questionnaires in secure databases and locked file cabinets in locked offices, password protection, and procedures for transferring study data to the secure password-protected cloud-based folder.
- All data collected in the study will be anonymized prior to sharing with the research team.
- Participant data for contacting participants for follow up will always be kept separate from interview responses. The de-identified data cannot be linked back to individual participants except through this log sheet, accessible only by Principal Investigator authorization. No subjects will be identified in any report or publication of the study or its results.

**Dissemination Policy:**Plans for investigators and sponsor to communicate trial results to participants, healthcare professionals, the public, and other relevant groups.

- We anticipate the results of our study will be of high interest to global public health planners, implementers, and donors who are seeking youth-friendly, innovative approaches to reach youth, change behavior, and impact their long-term health and well-being.
- We will work with the Rwandan Ministries, other key stakeholders, the school leadership staff and Society for Family Health to ensure that the results of our research will reach priority audiences in Rwanda, including other offices at the national and regional levels and health worker and educational professional associations. For international audiences, Dr. McCoy will work with the office of public relations at University of California, Berkeley (UCB), the UCB School of Public Health’s media department, and the UCB Center for Global Public Health to identify media platforms for reaching different target audiences.
- We plan to publish all main analyses peer-reviewed papers. We will also post results to [clinicaltrials.gov](http://clinicaltrials.gov/) within 90 days of publication of the results in a peer-reviewed journal. In addition, we will present the results at several forums, such as UCB global health meetings and small group meetings with in-country target audiences.
